# Field plus lab experiments help identify freezing tolerance and associated genes in subtropical evergreen broadleaf trees: A case study of *Camellia oleifera*

**DOI:** 10.3389/fpls.2023.1113125

**Published:** 2023-02-22

**Authors:** Haoxing Xie, Jian Zhang, Junyong Cheng, Songzi Zhao, Qiang Wen, Ping Kong, Yao Zhao, Xiaoguo Xiang, Jun Rong

**Affiliations:** ^1^Jiangxi Province Key Laboratory of Watershed Ecosystem Change and Biodiversity, Center for Watershed Ecology, Institute of Life Science and School of Life Sciences, Nanchang University, Nanchang, China; ^2^Hubei Provincial Engineering Research Center of Non-Timber Forest-Based Economy, Hubei Academy of Forestry, Wuhan, China; ^3^Jiangxi Provincial Key Laboratory of Camellia Germplasm Conservation and Utilization, Jiangxi Academy of Forestry, Nanchang, China; ^4^Jiangxi Ecological Meteorology Centre, Nanchang, China; ^5^Lushan Botanical Garden, Chinese Academy of Sciences, Lushan, China

**Keywords:** *Camellia*, cold-stress response, gene expression, lignin, signal transduction pathways, transcriptome, trees

## Abstract

The molecular mechanisms of freezing tolerance are unresolved in the perennial trees that can survive under much lower freezing temperatures than annual herbs. Since natural conditions involve many factors and temperature usually cannot be controlled, field experiments alone cannot directly identify the effects of freezing stress. Lab experiments are insufficient for trees to complete cold acclimation and cannot reflect natural freezing-stress responses. In this study, a new method was proposed using field plus lab experiments to identify freezing tolerance and associated genes in subtropical evergreen broadleaf trees using *Camellia oleifera* as a case. Cultivated *C. oleifera* is the dominant woody oil crop in China. Wild *C. oleifera* at the high-elevation site in Lu Mountain could survive below −30°C, providing a valuable genetic resource for the breeding of freezing tolerance. In the field experiment, air temperature was monitored from autumn to winter on wild *C. oleifera* at the high-elevation site in Lu Mountain. Leave samples were taken from wild *C. oleifera* before cold acclimation, during cold acclimation and under freezing temperature. Leaf transcriptome analyses indicated that the gene functions and expression patterns were very different during cold acclimation and under freezing temperature. In the lab experiments, leaves samples from wild *C. oleifera* after cold acclimation were placed under −10°C in climate chambers. A cultivated *C. oleifera* variety “Ganwu 1” was used as a control. According to relative conductivity changes of leaves, wild *C. oleifera* showed more freezing-tolerant than cultivated *C. oleifera*. Leaf transcriptome analyses showed that the gene expression patterns were very different between wild and cultivated *C. oleifera* in the lab experiment. Combing transcriptome results in both of the field and lab experiments, the common genes associated with freezing-stress responses were identified. Key genes of the flg22, Ca^2+^ and gibberellin signal transduction pathways and the lignin biosynthesis pathway may be involved in the freezing-stress responses. Most of the genes had the highest expression levels under freezing temperature in the field experiment and showed higher expression in wild *C. oleifera* with stronger freezing tolerance in the lab experiment. Our study may help identify freezing tolerance and underlying molecular mechanisms in trees.

## 1 Introduction

Cold stress is one of the common abiotic stresses that affects the growth, development and geographical distribution of plants, and the productions of crops (Lichtenthaler, 1998; Pearce, 2001). Based on temperature and damage on plants, cold stress is usually divided into chilling stress (0~15°C) and freezing stress (<0°C) (Liu et al., 2018a). Chilling stress induces rigidification of membranes in plant cells, changes protein conformation or disrupts the stability of protein complexes, accelerates the accumulation of reactive oxygen species (ROS), and affects photosynthesis (Ruelland et al., 2009). Freezing stress comes with extracellular ice crystals growth, which causes severe dehydration of cells and direct mechanical damage to cells (Pearce, 2001). Compared with chilling stress, freezing stress causes more serious damages to plants and even leads to plant death.

Plants have evolved a series of regulatory mechanisms to reduce the damage of cold stress. Plants develop greater tolerance to freezing stress after exposure to chilling temperature for a period of several days or weeks, a process known as cold acclimation (Thomashow, 1999). The molecular mechanisms of cold acclimation have been extensively and intensively studied in plants, especially in annual herbaceous plants (e.g. Arabidopsis, rice). Numerous genes have been identified in the process of cold acclimation, and their expression are induced by chilling temperature. During cold acclimation, the ICE1-CBF-COR transcriptional cascade is the most well-studied signaling pathway (Shi et al., 2015), which regulates the synthesis of antifreeze proteins and various protective substances (Ding et al., 2019). In addition, many signal molecules (e.g. Ca^2+^, H_2_O_2_), transcription factors (e.g. WRKY, AP2/EREBP), plant hormones (e.g. gibberellin, abscisic acid) and other substances have been confirmed to play an important role in cold acclimation (Shi et al., 2015; Ding and Yang, 2022).

Annual herbaceous plants usually complete their life cycles before harder winters. On the other hand, perennial woody plants overwinter with prolonged exposure to freezing stress and some can survive under much lower freezing temperatures than annual herbaceous plants, especially in high-latitude or high-elevation areas (Strimbeck et al., 2015). The molecular mechanisms of freezing tolerance in perennial woody plants may be more complex than in annual herbaceous plants (Wisniewski et al., 2014). The freezing tolerance processes in perennial woody plants may involve a suit of special mechanisms in addition to the well-studied cold acclimation processes in annual herbaceous plants. Considering the huge diversity within and between woody plant species widely distributed in varied climate zones, there are still many unsolved mysteries in the molecular mechanisms of freezing tolerance in woody plants.

Subtropical evergreen broadleaf woody plants are usually sensitive to freezing stress. One exception is wild *Camellia oleifera*, a long-lived evergreen broadleaf shrub or small tree. *Camellia oleifera* has a special phenology that it blooms in autumn and bears fruits overwinter. As one of the representative plant species in subtropical evergreen broadleaf forests, wild *C. oleifera* is widely distributed in the subtropical mountain and hilly areas in the Yangtze River Basin and the Southern China, with elevation ranging from about 200 to 2000 m (Ming, 2000; Zhuang, 2008). Wild *C. oleifera* showed rich genetic diversity and clear genetic differentiation among populations from different latitudes and longitudes with diverse climatic conditions (Cui et al., 2022). The wild *C. oleifera* population in Lu Mountain was found to have the most distinguished genetic structure (Cui et al., 2022). Lu Mountain is located in the north of Jiangxi Province, at the border between the middle and northern subtropical regions in China, and it is in the northern distribution periphery of wild *C. oleifera*. Adaptation isolation by cold climate conditions together with geographical isolation might lead to the distinct genetic structure of the wild *C. oleifera* population in Lu Mountain. The air temperature at high-elevation areas of Lu Mountain is generally around −10°C in winter, and even below −30°C to the extreme. Therefore, wild *C. oleifera* at high-elevation areas of Lu Mountain should be tolerant to deep freezing stress, which can be an ideal case for studying freezing tolerance and associated genes in evergreen broadleaf trees.

Under global climate change, extreme temperature events have increasingly exhibited around the world (Horton et al., 2015). Temperature patterns are gradually becoming irregular, leading to unexpectedly unusual freezing temperatures, which increase the risk of frostbite on crops (Pagter and Arora, 2013). An investigation report showed that freezing stress had serious impacts on the productions of economic forests in the middle and northern subtropical regions of China, especially for evergreen broadleaf trees such as cultivated *C. oleifera* (Yao and Wang, 2008). Cultivated *C. oleifera* is the dominant woody oil crops in China. The seed oil of *C. oleifera* is rich in oleic acid with up to >80%, known as “oriental olive oil” (Zhuang, 2008; Ma et al., 2011). It is urgent to breed cultivated *C. oleifera* varieties with strong freezing tolerance. Crop wild relatives are essential genetic resources for crop breeding, especially for increasing the tolerance to abiotic stresses (Mammadov et al., 2018). Thus, the studies for identifying freezing tolerant wild *C. oleifera* and underlying molecular mechanisms can facilitate the discovery and utilization of wild genetic resources for the breeding of cultivated *C. oleifera* with strong freezing tolerance.

Various methods have been used to evaluate freezing tolerance in temperate fruit trees under field or lab conditions (Yu and Lee, 2020). Since natural conditions involve many factors and temperature usually cannot be controlled, field experiments alone cannot directly identify the effects of freezing stresses. In labs, many studies used isolated leaves, shoots or flower buds and placed samples under controlled freezing temperatures of various durations in growth chambers or bath circulators (Yu and Lee, 2020). Then, to evaluate freezing tolerance, physiological and biochemical indicators are measured such as the changes in relative conductivity (REC) and contents of malondialdehyde, proline, soluble sugar, and soluble protein (Liu et al., 2013; Su et al., 2015). Under freezing stress, plant cell membrane is easy to rupture, and then cell contents seep out resulting in the increase of REC. Because REC can be estimated simply and rapidly, REC is the most frequently used estimate for evaluating tissue injury under freezing stress (Dong et al., 2002; Yu and Lee, 2020). In addition, studies have shown that under freezing stress changes in REC is correlated with changes in contents such as malondialdehyde, proline and soluble sugars (Cheng et al., 2017). On the other hand, freezing tolerance should be evaluated after plants have completed cold acclimation (Dong et al., 2002). However, lab conditions such as climate chambers usually are not sufficient for completing cold acclimation in evergreen trees, leading to weak freezing tolerance (Liu et al., 2020). Field conditions such as light signals and air temperature changes in autumn and winter may be essential for completing cold acclimation in evergreen trees (Liu et al., 2020). Gene expression patterns and enriched pathways may be different between the processes of field and lab cold acclimation (Liu et al., 2020). Freezing tolerance evaluation under lab conditions may not reflect the actual freezing tolerance processes under natural conditions (Yu and Lee, 2020). Therefore, a proper combination of field and lab experiments may help identify freezing tolerance and underlying molecular mechanisms in trees.

In this study, we designed field plus lab experiments to identify freezing tolerance and associated genes in an evergreen broadleaf tree *C. oleifera* with strong freezing tolerance. In the field experiment, temperature was monitored continually from autumn to winter on wild *C. oleifera* at a high-elevation site in Lu Mountain. Twigs of spring shoots were sampled at different periods (before cold acclimation, during cold acclimation and under freezing temperature). Some leaves were frozen immediately in liquid nitrogen for transcriptome analyses. In the lab experiment, leaf samples after cold acclimation from the field experiment were placed under −10°C in a climate chamber. As a control, leaf samples after cold acclimation from a commonly cultivated *C. oleifera* in the north of Jiangxi Province were also used in the lab experiment. Changes in REC of leaf samples through time were analyzed to evaluate the freezing tolerance of *C. oleifera*. Some leaf samples from the lab experiment were also used for transcriptome analyses. Combining the transcriptome analyses of leaf samples from the field and lab experiments, genes associated with freezing tolerance were identified in wild *C. oleifera*. This study demonstrates that the field plus lab experiments can help identify freezing tolerance and associated genes in evergreen broadleaf trees. Especially, the methods can help identify freezing tolerant wild *C. oleifera* and associated genes for the breeding of cultivated *C. oleifera* with strong freezing tolerance. This study can also improve the understanding of the molecular mechanisms of freezing tolerance in perennial woody plants.

## 2 Materials and methods

### 2.1 Field experiment

**Figure 1:**
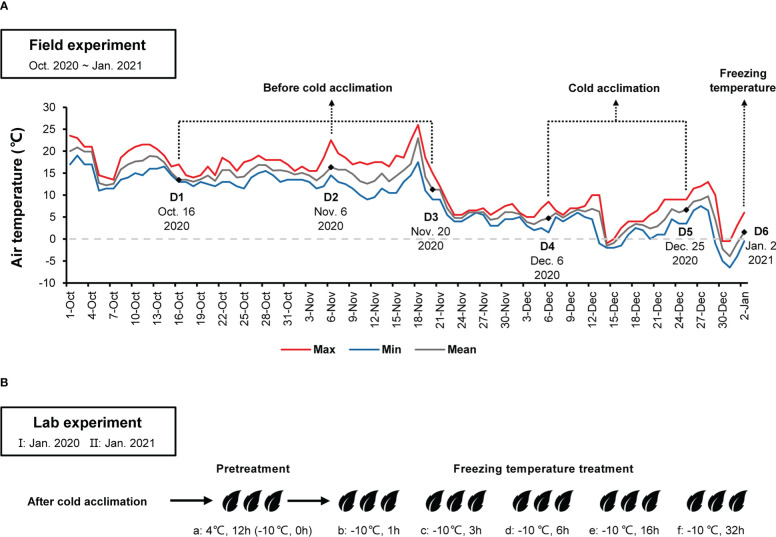
**(A)** Maximum, minimum, and mean air temperatures at the high-elevation site in Lu Mountain from October 2020 to January 2021. D1~D6 indicate sampling time in the field experiment for transcriptome sequencing. **(B)** Leaf samples after cold acclimation from the field experiment were placed in a climate chamber with pretreatment at 4°C for 12 h **(a)**, −10°C for 1 h **(b)**, 3 h **(c)**, 6 h **(d)**, 16 h **(e)** and 32 h **(f)**. Samples were taken for determining relative conductivity and transcriptome sequencing.

Field experiment was performed at a wild *C. oleifera* distribution site (29.583580°N, 115.985116°E, 993.47m) in Lu Mountain, Jiangxi Province, China. Three well-growing wild *C. oleifera* (LSG1~3) were selected. In January 2020, fresh twigs of spring shoots after cold acclimation were sampled from wild *C. oleifera* for the lab experiment I. From October 2020 to January 2021, a Thermochron iButton device DS1921G (Maxim Integrated, San Jose, CA, U.S.) was installed on a wild *C. oleifera* to monitor and record air temperature. Chen et al. (2017) found that wild *C. oleifera* undergone cold acclimation when air temperatures were below 10°C. Thus, twigs of spring shoots were sampled at different periods depending on the mean air temperature (≥10°C before cold acclimation, 0~10°C during cold acclimation and <0°C under freezing temperature) (**Figure 1A**). Some leaves were covered with aluminum foil and immediately placed in a vacuum bottle with liquid nitrogen, then stored at −80°C refrigerator in the lab for subsequent transcriptome analyses. Fresh twigs of spring shoots after cold acclimation were also used for the lab experiment II.

A commonly cultivated *C. oleifera* variety “Ganwu 1” (GW1) in the north of Jiangxi Province was used as a control. GW1 is an excellent clone line bred by Jiangxi Academy of Forestry in 2007. GW1 is suitable for growing in low mountains and hills at altitudes below 100 m in Jiangxi Province. In January 2020 and 2021, for the lab experiment I and II, fresh twigs of spring shoots after cold acclimation were sampled from three clones of GW1 in the *Camellia* Gene Bank, Jiangxi Academy of Forestry, Nanchang, Jiangxi Province, China. Air temperature data of Jiangxi Academy of Forestry were obtained from Jiangxi Ecological Meteorology Centre.

### 2.2 Lab experiment

At least twelve fresh and undamaged leaves with petioles were collected from twigs of each tree at the same day of sampling. Surface of leaves are cleaned with deionized water. One to three leaves of the same tree were packed in a perforated plastic ziplock bag (120 mm × 85 mm). For pretreatment, leaves were placed in a dark 4°C climate chamber RXZ-0358-LED (Ningbo Jiangnan Instrument Factory, Ningbo, China) for 12 hours. Afterwards, some leaves were taken to determine the REC before freezing temperature treatment.

For freezing temperature treatment, bags of leaves were transferred to a dark −10°C climate chamber after pretreatment. Samples were placed in the −10°C climate chamber for 1 h, 3 h, 6 h, 16 h and 32 h, respectively (**Figure 1B**). After freezing temperature treatment, samples were thawed in a dark 0°C climate chamber for 12 h. Then, REC of leaves was determined.

For the lab experiment II in January 2021, except for the leaves used for REC determination, the remaining leaves of the same treatment were covered by aluminum foil and immediately placed in a vacuum bottle with liquid nitrogen, then stored at −80°C refrigerator in the lab for subsequent transcriptome sequencing.

As controls, after pretreatment, bags of leaves from LSG and GW1 were placed in the dark 4°C climate chamber, and REC of leaves was determined at 6 h and 32 h, respectively. In addition, bags of leaves from LSG and GW1 before cold acclimation were used for comparison: pretreatment at dark 4°C for 12 h; freezing temperature treatment at dark −10°C for 1 h, 3 h, 6 h, 16 h and 32 h, respectively; thawing at dark 0°C for 12 h and determining REC of leaves.

### 2.3 REC determination and analysis

Each leaf was punched avoiding veins with a hole puncher to obtain five wafers (5 mm in diameter). Wafers of each leaf were placed into a clean 50 mL tube, and then 20 mL deionized water was added to the tube. The sample tube was placed at room temperature for 24 h. The conductivity of the solution in the sample tube was determined using a conductivity meter FE38 (Mettler Toledo, Shanghai, China), recorded as C_1_. Afterwards, the solution in the sample tube was boiled (105°C for 20 min) in an autoclave SX-500 (Tomy Kogyo, lshikawagun, Fukushima, Japan), and the conductivity of the solution was determined after cooling to room temperature, recorded as C_2_. The conductivity of deionized water used was recorded as C_0_. Finally, REC of leaves was calculated based on the following equation:


$$REC\left(\%\right)=\left[\frac{\left(C_{1}-C_{0}\right)}{\left(C_{2}-C_{0}\right)}\right]\times 100\%$$

Statistical analysis of leaf REC was performed in IBM SPSS (v25.0). The analysis of variance (ANOVA) was applied to determine significant differences among treatments (*p *< 0.05).

### 2.4 RNA extraction, transcriptome sequencing, and unigene annotation

Total RNA was extracted from each leaf sample using the EASYspin Plus Plant RNA Kit (Aidlab, Beijing, China) according to the manufacturer’s instructions. RNA samples concentration and quality were determined using a NanoDrop 2000 spectrophotometer (Thermo Fisher Scientific, Waltham, MA, USA) and an Agilent Bioanalyzer 2100 system (Agilent Technologies, Palo Alto, CA, USA). High-quality RNA samples (OD260/280 ≥ 1.8 and OD260/230 ≥ 1.8) were used to construct cDNA libraries for transcriptome sequencing at Beijing Genomics Institute (BGI, Wuhan, China).

For RNA-sequencing (RNA-seq), in the field experiment, each sampling time was considered one treatment, and each treatment included three biological replicates (LSG1~3); in the lab experiment II, each treatment included three biological replicates (LSG1~3 and three clones of GW1). Paired-end sequencing (2 × 150 bp) was carried out on the MGISEQ-2000 platform (BGI, Wuhan, China). Clean reads were obtained by removing reads containing adapter and/or ploy-N and/or low qualified reads from raw reads by SOAPnuke (v1.5.2) (Cock et al., 2010).

For Isoform-sequencing (ISO-seq), in the field experiment and lab experiment II, sample of at least one biological replicate from each treatment was taken and all the samples were mixed together. Sequencing was carried out on the PacBio Sequel II platform (Pacific Biosciences, Menlo Park, CA, USA). Subreads obtained after sequencing were processed using the SMRT Link software (v8.0), including reads of insert (ROI), reads classification, reads clustering and correction to obtain high quality full-length consensus sequences (isoforms). Then, all isoforms were merged and the redundant sequences were removed from isoforms using CD-HIT software (v4.8.1) to obtain unigenes (Fu et al., 2012). Finally, evaluation of transcriptome sequencing data integrity was performed with Benchmarking Universal Single-Copy Orthologs (BUSCO) software (v3.0.1). Because there is no reference genome for *C. oleifera*, all unigenes resulted from the ISO-seq constituted the reference transcriptome used for RNA-seq.

Functional annotations of all unigenes were based on one or more of the following databases: NCBI non-redundant nucleotide sequences (NT), NCBI non-redundant protein sequences (NR), euKaryotic Ortholog Groups (KOG), Kyoto Encyclopedia of Genes and Genomes (KEGG), Swiss-Prot, Protein family (Pfam) and Gene Ontology (GO). Blast (v2.2.23) and Diamond (v0.8.31) were used for NT, NR, KOG, KEGG and Swiss-Prot annotations (Altschul et al., 1990; Buchfink et al., 2015). The hmmscan in HMMER (v3.0) was used to perform Pfam annotations (Finn et al., 2011). The GO annotations were performed with Blast2GO (v2.5.0) based on the NR annotations (Conesa et al., 2005).

### 2.5 Gene expression analysis

Clean reads from RNA-seq were aligned to the reference transcriptome using Bowtie2 (v2.2.5). Read count and expression level of each gene in a sample were calculated using RSEM (v1.3.3) (Li and Dewey, 2011). FPKM (expected number of fragments per kilobase of transcript per million fragments mapped) (Trapnell et al., 2010) was used to evaluate the expression level of genes. To compare differences in gene expression patterns of samples, the prcomp function in R software was used to perform principal component analysis (PCA) on the FPKM data of all genes in samples from the field experiment and lab experiment II, respectively.

Differential gene expression was analyzed using the R package DESeq2 (v1.26.0) based on the principle of negative binomial distribution (Love et al., 2014). Gene expression was considered to be significantly different with *Q*-value (Adjusted *P*-value)< 0.05 and such a gene was a differentially expressed gene (DEG). The R package VennDiagram (v1.7.3) was used to make Venn Diagram of DEGs among samples.

### 2.6 Weighted gene co-expression network analysis

The R package WGCNA (v1.70.3) was employed to construct co-expression network (Langfelder and Horvath, 2008). The FPKM data of all genes in samples from the field experiment were used as inputs for the co-expression network analysis. The correlation matrix was transformed into an adjacency matrix by setting the soft threshold to 22 (**Figure S1**). The topological overlap matrix (TOM) was transformed from an adjacency matrix using the dissimilarity calculation method, and then a clustering tree was built. Gene modules were obtained based on the standard of dynamic tree cut algorithm, and the parameter settings were minModuleSize = 30 and MEDissThres = 0.4. Correlations analysis was performed between gene modules and sampling time to screen the gene modules associated with cold acclimation or freezing temperature responses in the field experiment. Cytoscape (v3.9.1) was used to construct co-expression network maps and screen hub genes.

### 2.7 KEGG pathway enrichment analysis and time-series analysis

According to the KEGG annotation results, the phyper function in R software was used for KEGG pathway enrichment analysis of the gene modules identified in WGCNA of samples from the field experiment as well as DEGs between LSG and GW1 from the lab experiment II, respectively. Pathway with *Q*-value (Adjusted *P*-value)< 0.05 were considered to be significantly enriched by genes, *Q*-value< 0.01 were considered to be highly significantly enriched by genes, *Q*-value< 0.001 were considered to be very highly significantly enriched by genes. The R package Mfuzz (v2.34.0) was used to conduct the time-series analysis for the FPKM data of all genes in the field experiment (Kumar and Futschik, 2007).

Genes were compared between the gene modules associated with freezing temperature responses in the field experiment and the DEGs between LSG and GW1 under freezing temperature treatments in the lab experiment II. The intersection of both was selected and used for the KEGG pathway enrichment analysis. Expression level cluster heat map of the genes was made using the R package pheatmap (v1.0.12).

## 3 Results

### 3.1 Air temperature at the field experimental site

From October 2020 to January 2021, the air temperature at the field experimental site changed dramatically (**Figure 1A**). Before 21 November 2020, the mean air temperature was above 10°C. Afterwards, the mean air temperature dropped to around 5°C and fluctuated up and down. On 31 December 2020, the minimum air temperature dropped down to about −6°C. According to the air temperature changes at the field experimental site, sampling time in the field experiment were divided into three periods: D1, D2 and D3 before cold acclimation, D4 and D5 during cold acclimation, and D6 under freezing temperature (**Figure 1A**). The variation trends of the minimum air temperature one week before D6 were similar between the field experimental site in Lu Mountain and the *Camellia* Gene Bank of Jiangxi Academy of Forestry (**Figure S2**). In the *Camellia* Gene Bank of Jiangxi Academy of Forestry, the minimum air temperature dropped down to −3.3°C on January 1, 2021. Samples were collected from GW1 in the *Camellia* Gene Bank on January 2, 2021 (the same date of D6 in the field experiment) and used in the lab experiment II.

### 3.2 Evaluation of freezing tolerance in the lab experiment I and II

**Figure 2:**
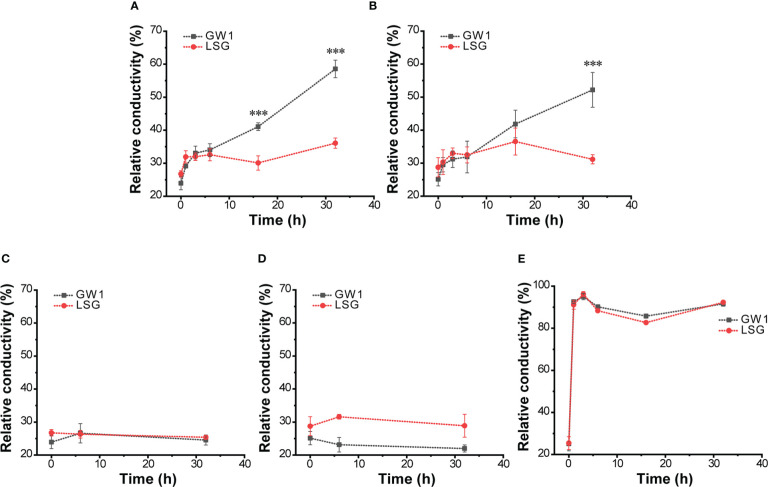
Changes of relative conductivity in: **(A)** leaf samples at −10°C in the lab experiment I; **(B)** leaf samples at −10°C in the lab experiment II; **(C)** leaf samples at 4°C controls in the lab experiment I; **(D)** leaf samples at 4°C controls in the lab experiment II; and **(E)** leaf samples (before cold acclimation) at −10°C in the lab experiment. The symbol *** indicates that there is significant difference (*p* < 0.01) between treatments in the analysis of variance.

Under the −10°C freezing temperature treatment, the leaf REC of GW1 increased with the increase in treatment time while the leaf REC of LSG only slightly fluctuated (**Figures 2A, B**). For 1~6 h at −10°C, the leaf REC of both LSG and GW1 increased slightly with no significant difference between them (*p* > 0.05). For a longer time at −10°C, the difference in the leaf REC became larger between LSG and GW1. As the −10°C freezing temperature treatment time reached 32 h, the leaf REC of GW1 exceeded 50% (I: 58.57%, II: 58.85%), significantly higher (*p* < 0.01) than the leaf REC of LSG (I: 36.07%, II: 36.63%). As controls, under the 4°C treatment for 32 h, leaf REC of both LSG and GW1 kept stable (I: both about 25%, II: GW1 about 25% and LSG about 30%) with no significant difference between each other (**Figures 2C–D**), indicating that the isolated leaves of both LSG and GW1 remained fresh after being placed at 4°C for 32 h. The results were highly similar between the lab experiment I and II in Jan 2020 and Jan 2021 (**Figures 2A–D**). The results indicated that, during the −10°C freezing temperature treatment, the GW1 leaves suffered more serious damages than the LSG leaves, suggesting stronger freezing tolerance of LSG than that of GW1.

For the leaf samples before cold acclimation, the leaf REC of both LSG and GW1 increased rapidly to about 90% under the −10°C freezing temperature treatment for only 1 h, and then remained around 90% for up to 32 h (**Figure 2E**). The results indicated that, without cold acclimation, shortly after the −10°C freezing temperature treatment, leaves of both LSG and GW1 were severely damaged.

### 3.3 Summary of transcriptome sequencing and functional annotation

Transcriptome sequencing samples were obtained from the field experiment and lab experiment II. A total of 45.88 Gb subreads were obtained from ISO-seq, and finally 400457 unigenes were obtained with length ranging from 200 bp to 19005 bp. The total length of the unigenes was about 500 Mb, the N50 value was 1667 bp and the N90 value was 666 bp. The evaluation results of BUSCO showed that more than 90% of sequences matched the BUSCO database (**Figure S3**). In RNA-seq, a total of 54 samples were sequenced from the lab experiment II, and the average amount of data obtained for each sample was 6.45 Gb. The average alignment rate to the reference transcriptome was 78.22%, and 323719 genes were aligned. On the other hand, a total of 36 samples were sequenced from the field experiment, and the average amount of data obtained for each sample was 6.41 Gb. The average alignment rate to the reference transcriptome was 78.28%, and 317301 genes were aligned.

There were 353724 (88.33%), 311843 (77.87%), 226082 (56.46%), 229689 (57.36%), 224355 (56.02%), 191985 (47.94%), and 237311 (59.26%) unigenes annotated in the NT, NR, KOG, KEGG, Swiss-Prot, Pfam and GO databases, respectively. In sum, 109934 (27.45%) unigenes were annotated in all the databases, and 371208 (92.70%) unigenes were annotated in at least one of the databases used in our study.

### 3.4 PCA and differential gene expression analysis

#### 3.4.1 Samples of field experiment

**Figure 3:**
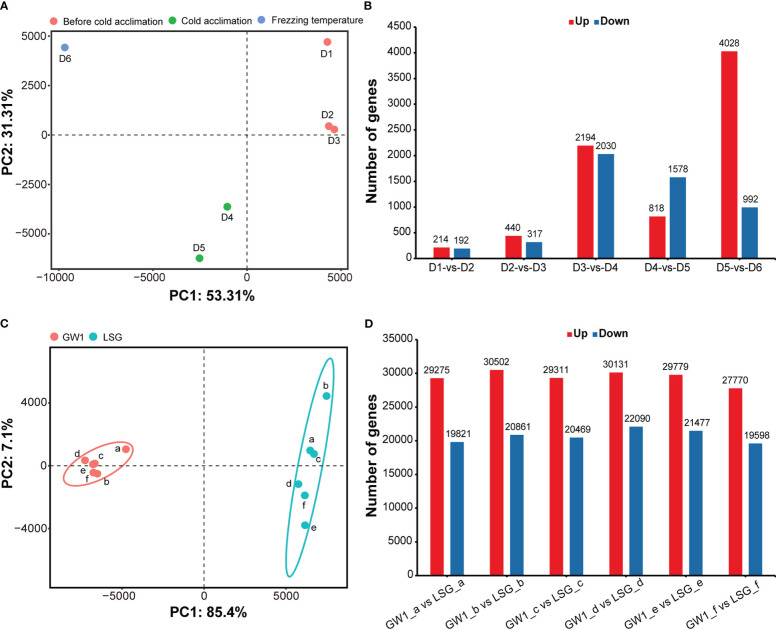
**(A)** Principal component analysis based on the FPKM data of all genes in the field experiment. **(B)** Number of differentially expressed genes between samples at adjacent sampling time in the field experiment. **(C)** Principal component analysis based on the FPKM data of all genes in the lab experiment II. **(D)** Number of differentially expressed genes between GW1 and LSG samples at each treatment time in the lab experiment II.

The results of PCA showed that samples were separated into three groups depending on three sampling time periods: 1) D1, D2 and D3 before cold acclimation; 2) D4 and D5 during cold acclimation; and 3) D6 under freezing temperature (**Figure 3A**). The results indicated that the gene expression patterns were very different among samples before cold acclimation, during cold acclimation and under freezing temperature.

Number of DEGs between adjacent sampling time showed that much more DEGs were found to be up-regulated between PCA groups than within PCA groups (**Figure 3B**). Especially, the number of up-regulated DEGs between D5 during cold acclimation and D6 under freezing temperature was the highest with up to 4028 up-regulated DEGs, almost two times of the number of up-regulated DEGs between D3 before cold acclimation and D4 during cold acclimation (**Figure 3B**), and only a few of DEGs are common to both (**Figure S4**). Such results indicated that after cold acclimation gene expression patterns may change dramatically under freezing stress.

#### 3.4.2 Samples of lab experiment II

In the results of PCA, samples were divided into two groups, GW1 samples were clustered into one group and LSG samples were clustered into another group (**Figure 3C**). The results indicated that gene expression patterns of LSG and GW1 were very different under freezing temperature treatment.

The results of differential gene expression analysis showed that a lot of DEGs (about 50000) occurred between LSG and GW1 samples at each treatment time (a, b, c, d, e, f), and a total of 88290 DEGs were found (**Figure 3D**). These DEGs may be associated with freezing tolerance, causing LSG to perform better than GW1 under freezing temperature treatment.

### 3.5 Construction of weighted gene co-expression networks

**Figure 4:**
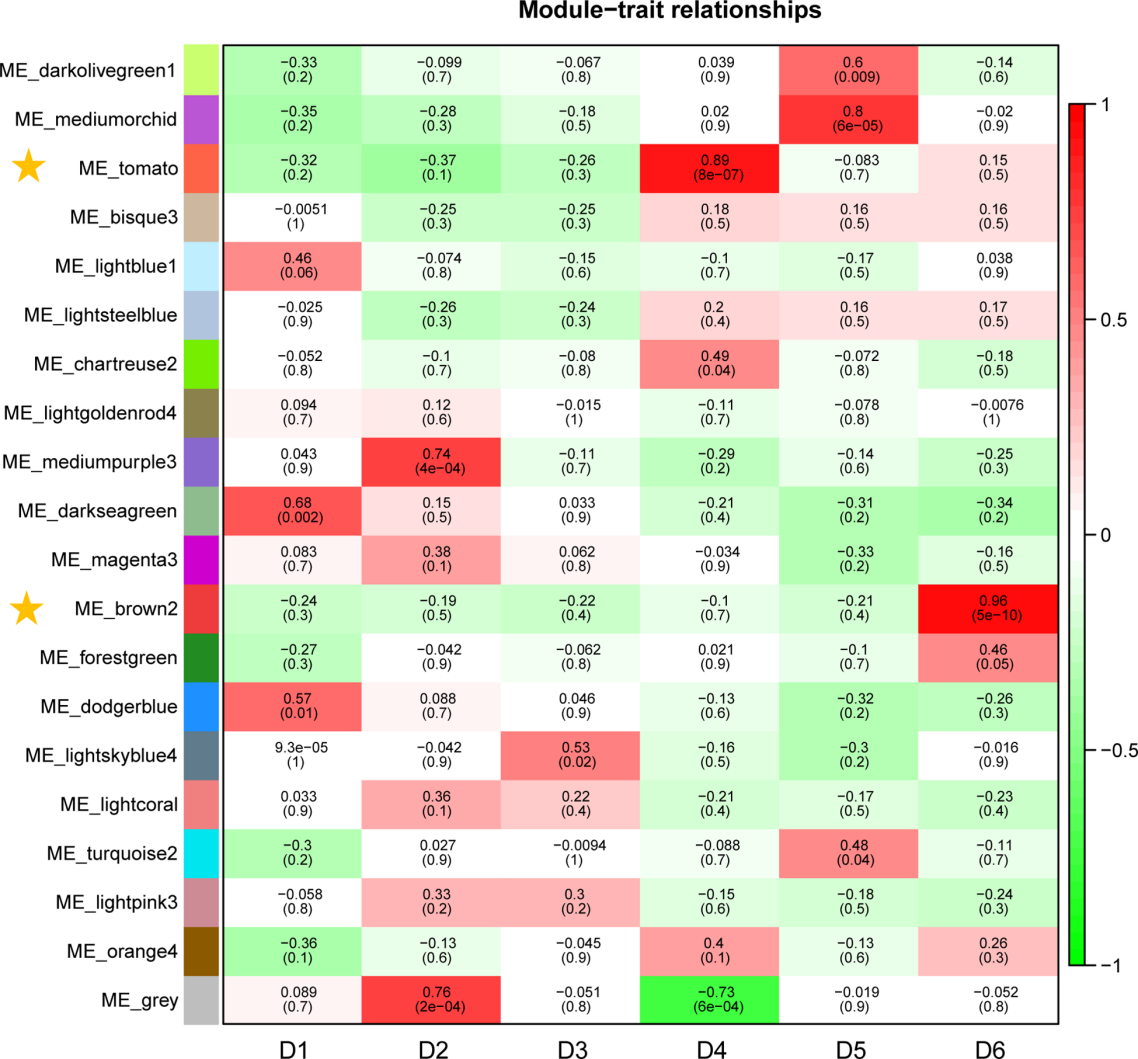
Correlations between gene module and sampling time in the field experiment. Each row corresponds to a module, and correlation coefficient values and *p*-values (in brackets) are indicated in cells.

In this study, WGCNA revealed 20 modules (**Figure S5**). Correlation analyses between modules and sampling time revealed that the ME_brown2 module had the highest *r* value (*r* = 0.96) and the lowest *p* value (*p* = 5E−10) in all the correlation analyses showing significantly positive correlation with D6 under freezing temperature (**Figure 4**). The ME_tomato module had the second highest *r* value (*r* = 0.89) and the second lowest *p* value (*p* = 8E−07) in all the correlation analyses showing significantly positive correlation with D4 during cold acclimation (**Figure 4**). The results suggested that the genes in the ME_tomato module may be involved in cold acclimation and the ME_brown2 module may be involved in responses to freezing temperature, and therefore were selected for further analysis. In addition, co-expression network was constructed using the top 100 hub genes with high connectivity (degree) from the ME_brown2 module (**Figure S6**).

### 3.6 KEGG pathway enrichment analysis and time-series analysis

#### 3.6.1 ME_tomato module and ME_brown2 module

**Figure 5:**
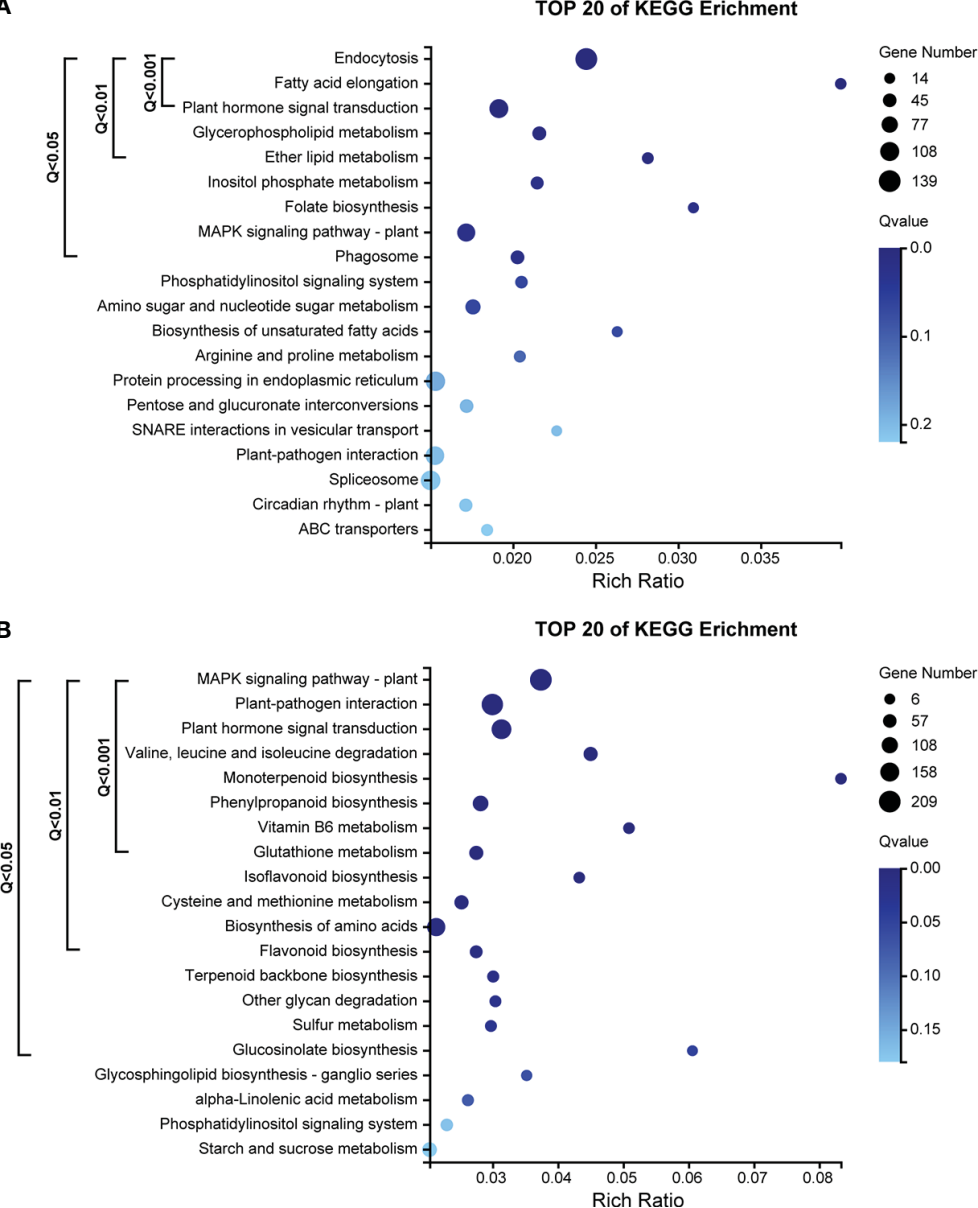
**(A)** KEGG pathway enrichment analysis of 4054 genes in the ME_tomato module, significantly correlated with D5 during cold acclimation in the field experiment. **(B)** KEGG pathway enrichment analysis of 5228 genes in the ME_brown2 module, significantly correlated with D6 under freezing temperature in the field experiment.

The ME_tomato module had a total of 4054 genes. The results of KEGG pathway enrichment analysis showed that 9 pathways were significantly enriched (Q-value< 0.05) by genes (**Figure 5A**), including 438 genes. Pathways with very highly significantly enriched (Q-value< 0.001) by genes were those involving in the ko04144 “endocytosis”, ko00062 “fatty acid elongation” and ko04075 “plant hormone signal transduction”, and there were 264 genes in these pathways (**Figure 5A**).

The ME_brown2 module had a total of 5228 genes. The results of KEGG pathway enrichment analysis showed that 16 pathways were significantly enriched (Q-value< 0.05) by genes (**Figure 5B**), including 969 genes. Pathways with very highly significantly enriched (Q-value< 0.001) by genes were those involving in the ko04016 “MAPK signaling pathway-plant”, ko04626 “plant-pathogen interaction”, ko04075 “plant hormone signal transduction”, ko00280 “valine, leucine and isoleucine degradation”, ko00902 “monoterpenoid biosynthesis”, ko00940 “phenylpropanoid biosynthesis”, ko00750 “vitamin B6 metabolism” and ko00480 “glutathione metabolism”, and there were 695 genes in these pathways (**Figure 5B**).

The time-series analysis divided the gene expression changes of the field experiment into 12 clusters (**Figure S7A**). In the ME_tomato module, among the 438 genes involving in the significantly enriched KEGG pathways, 258 genes were classified in the Cluster 4, 86 genes were classified into the Cluster 10, and 52 genes were classified into the Cluster 6, where gene expressions were clearly up-regulated in D4 during cold acclimation (**Figure S7B**). In the ME_brown2 module, among the 969 genes in the significantly enriched KEGG pathways, 754 genes were classified in the Cluster 6, 107 genes were classified in the Cluster 9, and 42 genes were classified in the Cluster 1, where gene expressions were clearly up-regulated in D6 under freezing temperature (**Figure S7B**).

In sum, the gene functions and expression patterns were different between the ME_tomato module correlated with D4 during cold acclimation and the ME_brown2 module correlated with D6 under freezing temperature.

#### 3.6.2 Genes in both field and lab experiments

A total of 88290 DEGs were found between GW1 and LSG samples under freezing temperature treatment from the lab experiment II. The results of KEGG pathway enrichment analysis of all the DEGs showed that three of the pathways with very highly significantly enriched by genes were the same as those in the ME_brown2 module from the field experiment, including “plant hormone signal transduction”, “MAPK signaling pathway-plant” and “plant-pathogen interaction” (**Figure S8**). However, the other pathways very highly significantly enriched by genes were different (**Figure S8**).

**Figure 6:**
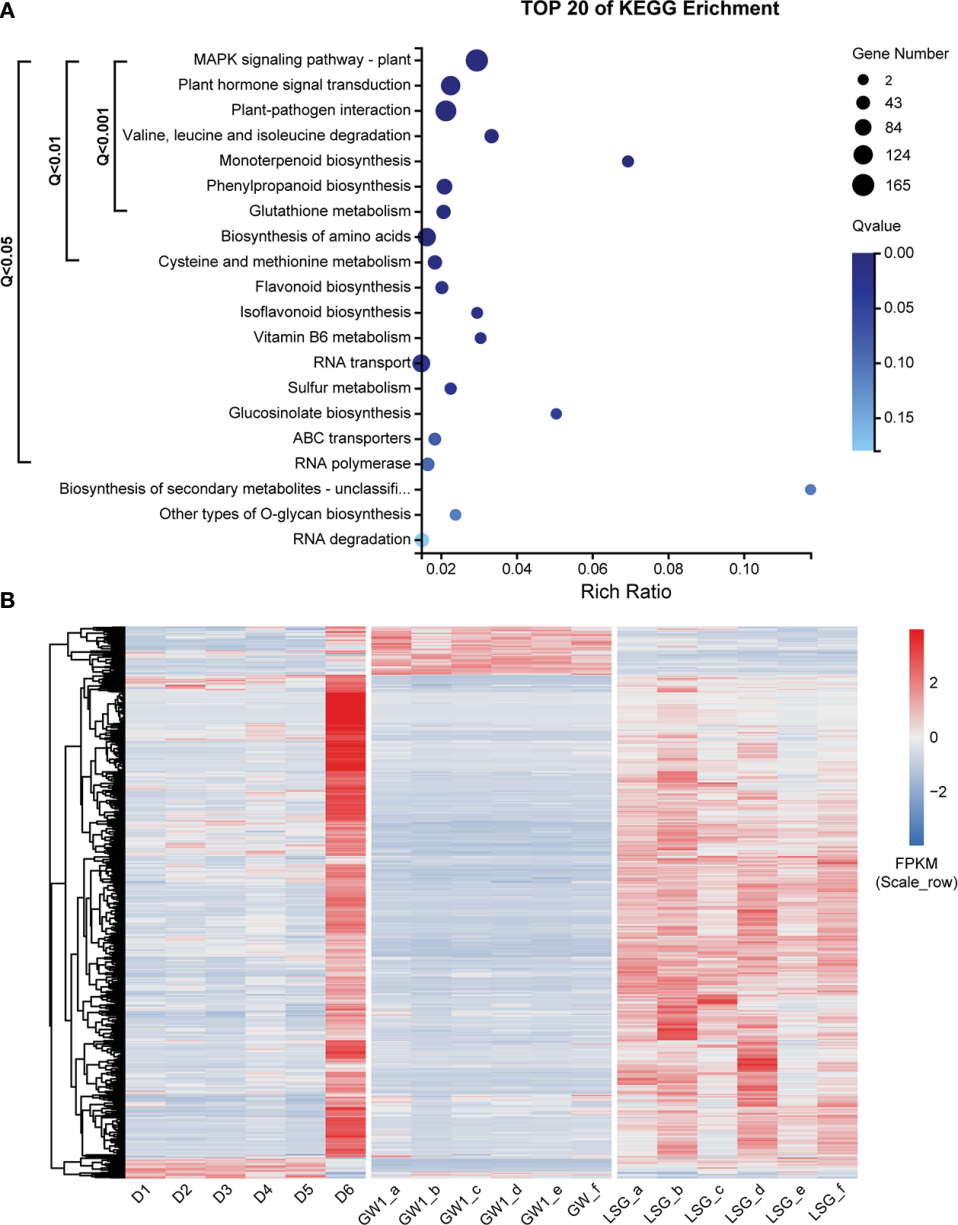
**(A)** KEGG pathway enrichment analysis of 3441 genes, the intersection of genes in the ME_brown2 module significantly correlated with D6 under freezing temperature in the field experiment and the differentially expressed genes between GW1 and LSG samples under −10°C in the lab experiment II. **(B)** Expression level cluster heat map of 503 genes that very highly significantly enriched in the KEGG pathway enrichment results of 3441 genes.

Comparing genes in the ME_brown2 module (5228 genes) from the field experiment and the DEGs (88290 genes) between GW1 and LSG samples from the lab experiment II, the intersection of both contained 3441 genes, 65.8% of the genes in the ME_brown2 module (field) and only 3.9% of the DEGs between GW1 and LSG (lab) (**Figure S9**). The results of KEGG pathway enrichment analysis of these genes showed that 15 pathways were significantly enriched (Q-value< 0.05) by genes, including 752 genes (**Figure 6A**). Pathways with very highly significantly enriched (Q-value< 0.001) by genes were those involving in the ko04016 “MAPK signaling pathway-plant”, ko04075 “plant hormone signal transduction”, ko04626 “plant-pathogen interaction”, ko00280 “valine, leucine and isoleucine degradation”, ko00902 “monoterpenoid biosynthesis”, ko00940 “phenylpropanoid biosynthesis” and ko00480 “glutathione metabolism” pathways, and there were 503 genes in these pathways (**Figure 6A** and **Table S1**). Moreover, 20 of the 503 genes were also among the top 100 hub genes in the ME_brown2 module (**Table S2**). In the 20 hub genes, 13 genes were annotated into “plant hormone signal transduction” pathway, 5 genes were annotated into “MAPK signaling pathway-plant” pathway, 2 genes were annotated into “plant-pathogen interaction” pathway, and 1 gene was annotated into “valine, leucine and isoleucine degradation” pathway. Such KEGG pathway enrichment results were more similar to those in the ME_brown2 module from the field experiment (**Figure 5B**) than those in the DEGs from the lab experiment (**Figure S8**). Expression level cluster heat map of the 503 genes showed that the expression levels of most genes were much higher at D6 under freezing temperature than in other periods of the field experiment. In the lab experiment II, most genes had higher expressions in LSG samples with strong freezing tolerance than those in GW1 samples with weak freezing tolerance (**Figure 6B**). Overall, the expression patterns of most genes were similar under freezing temperature between the field and lab experiments, and these genes may play an important role in the responses to freezing stress.

### 3.7 Gene expression patterns in important pathways associated with freezing tolerance in the field and lab experiments

#### 3.7.1 Signal transduction pathways

**Figure 7:**
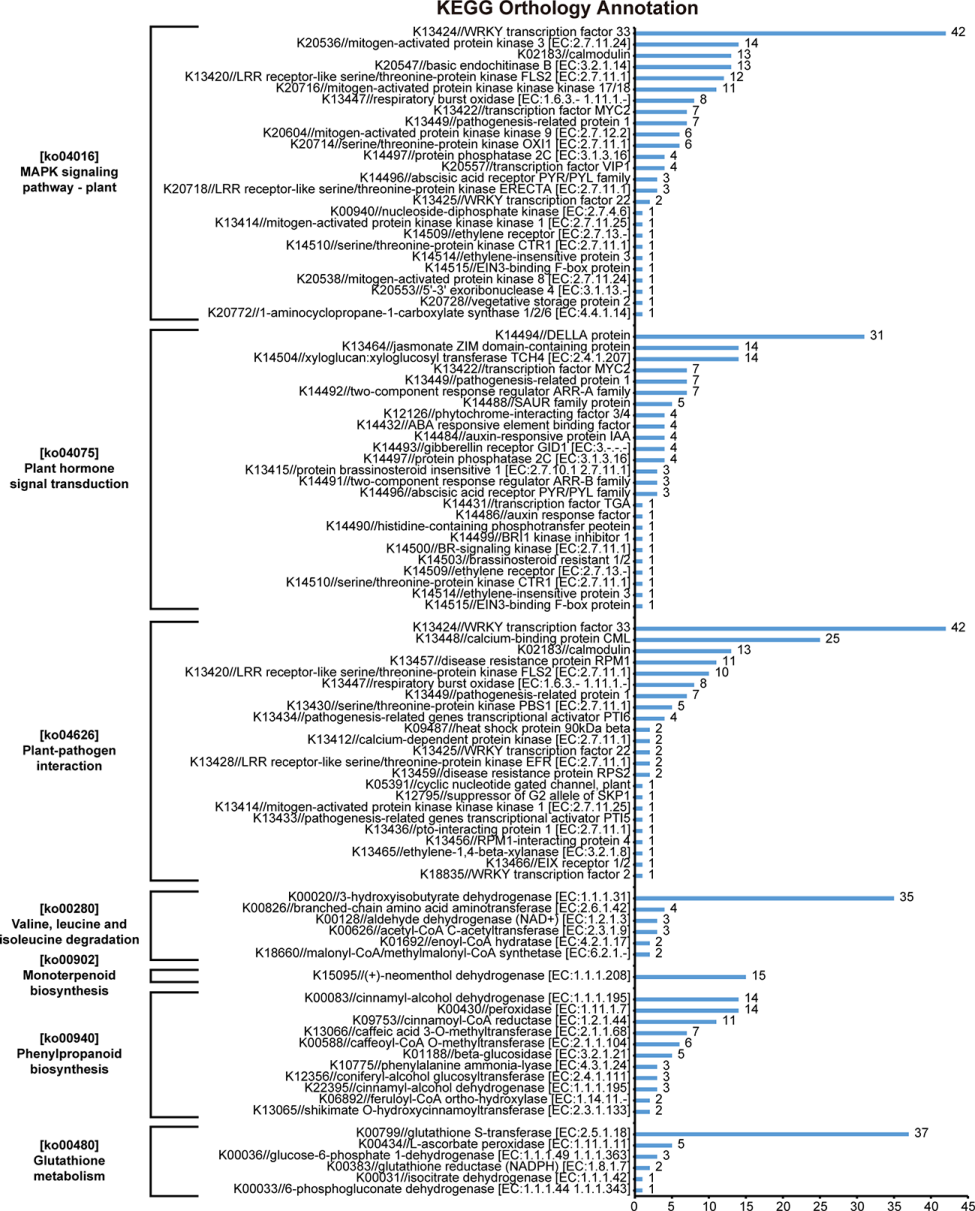
KEGG orthology annotation results of 503 genes that very highly significantly enriched in the KEGG pathway enrichment results of 3441 genes, the intersection of genes in the ME_brown2 module significantly correlated with D6 under freezing temperature in the field experiment and the differentially expressed genes between GW1 and LSG samples under −10°C in the lab experiment II.

**Figure 8:**
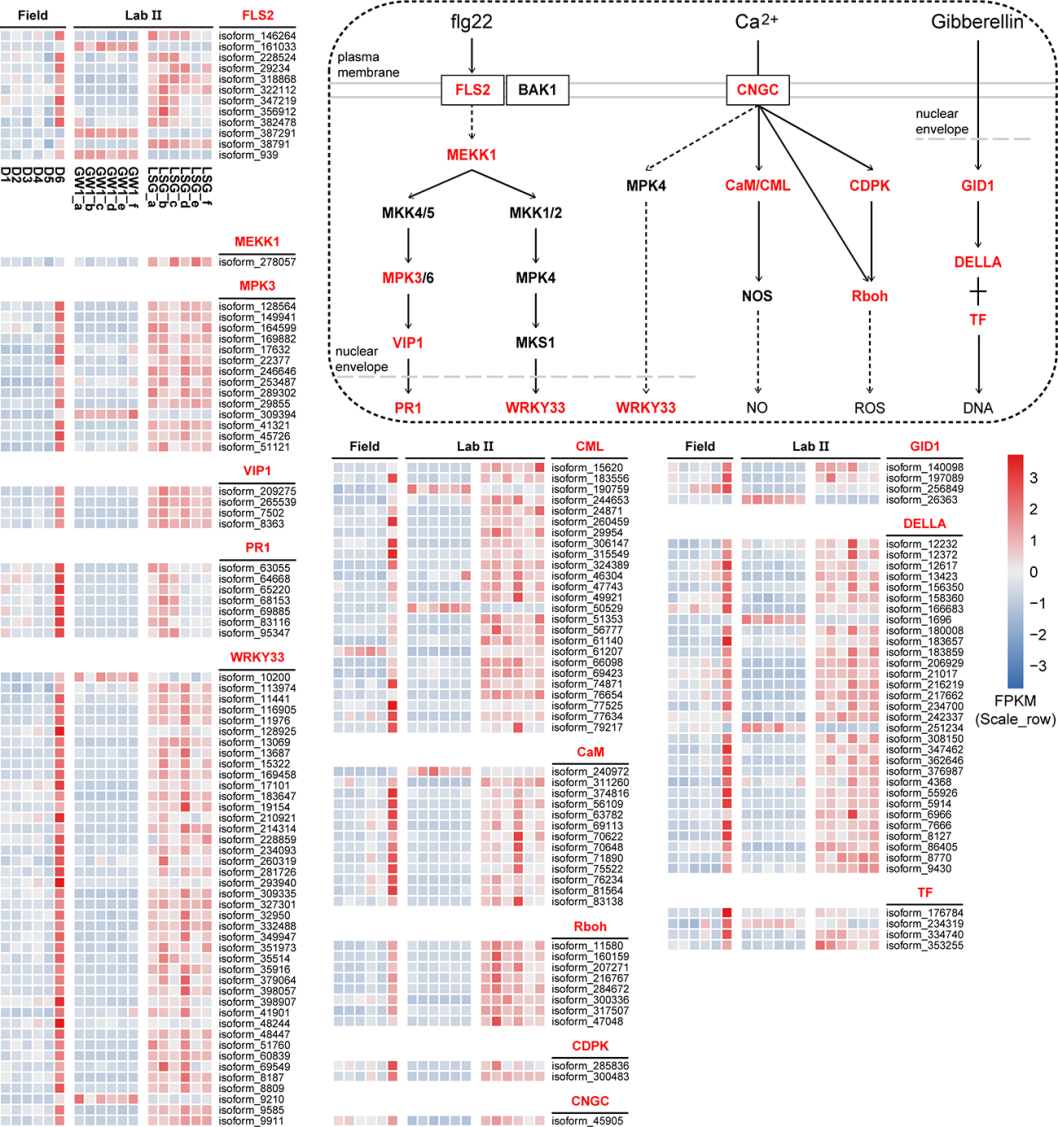
The flg22 signal transduction pathway in the ko04016 “MAPK signaling pathway-plant” pathway, the Ca^2+^ signal transduction pathway in the ko04626 “plant-pathogen interaction” pathway, the gibberellin signal transduction in the ko04075 “plant hormone signal transduction” pathway, and expression level cluster heat maps of key genes in these pathways in the field and lab experiments.

The results of KEGG orthology annotation of the 503 genes showed that 165 genes were divided into 26 functions in the ko04016 “MAPK signaling pathway-plant” pathway, and 42 genes were annotated as K13424 “WRKY transcription factor 33” (WRKY33) in this pathway (**Figure 7**). WRKY33 was closely related to the flagellin 22 (flg22) signal transduction pathway of this pathway. In addition to WRKY33, 38 genes were annotated into the flg22 signal transduction pathway, including K20536 “mitogen-activated protein kinase 3” (MPK3) with 14 genes, K13420 “LRR receptor-like serine/threonine-protein kinase FLS2” with 12 genes, K13449 “pathogenesis-related protein 1” (PR1) with 7 genes, K20557 “transcription factor VIP1” with 4 genes, and K13414 “mitogen-activated protein kinase kinase kinase 1” (MEEK1) with 1 gene (**Figure 7**). In the field experiment, the expression levels of most genes were low from D1 to D5 and were up-regulated to the highest at D6; in the lab experiment II, the expression levels of genes in LSG samples were usually higher than those in GW1 samples, only a few genes showed the opposite (**Figure 8**).

In the ko04626 “plant-pathogen interaction” pathway, 144 genes were divided into 23 functions, and 42 genes were also annotated as K13424 WRKY33 in this pathway. These WRKY genes were concentrated in the Ca^2+^ signal transduction pathway of this pathway. And there were many other genes in the Ca^2+^ signal transduction pathway (58 genes), including K13448 “calcium-binding protein CML” with 25 genes, K02183 “calmodulin” (CaM) with 13 genes, K13447 “respiratory burst oxidase” (Rboh) with 8 genes, K13412 “calcium-dependent protein kinase” (CDPK) with 2 genes, and K05391 “cyclic nucleotide gated channel, plant” (CNGC) with 1 gene (**Figure 7**). In the field experiment and lab experiment II, the expression patterns of most genes in the Ca^2+^ signal transduction pathway were similar to those of most genes in the flg22 signal transduction pathway (**Figure 8**).

In the ko04075 “plant hormone signal transduction” pathway, 124 genes were divided into 25 functions, and 31 genes were annotated as K14494 “DELLA protein” in this pathway, which is an important part of the gibberellin signal transduction pathway in this pathway. K14493 “gibberellin receptor GID1” and K12126 “phytochrome-interacting factor 3/4” (TF) also belong to the gibberellin signal transduction pathway, with 4 genes respectively (**Figure 7**). In the field experiment and lab experiment II, the expression patterns of most genes in the gibberellin signal transduction pathway were similar to those of most genes in the flg22 signal transduction pathway as well as in the Ca^2+^ signal transduction pathway (**Figure 8**).

In sum, expression levels of most genes in these signal transduction pathways were up-regulated to the highest at D6 under freezing temperature in the field experiment, and in the lab experiment, most genes showed higher expressions in LSG samples than those in GW1 samples (**Figure 8**).

#### 3.7.2 Lignin biosynthesis pathway

**Figure 9:**
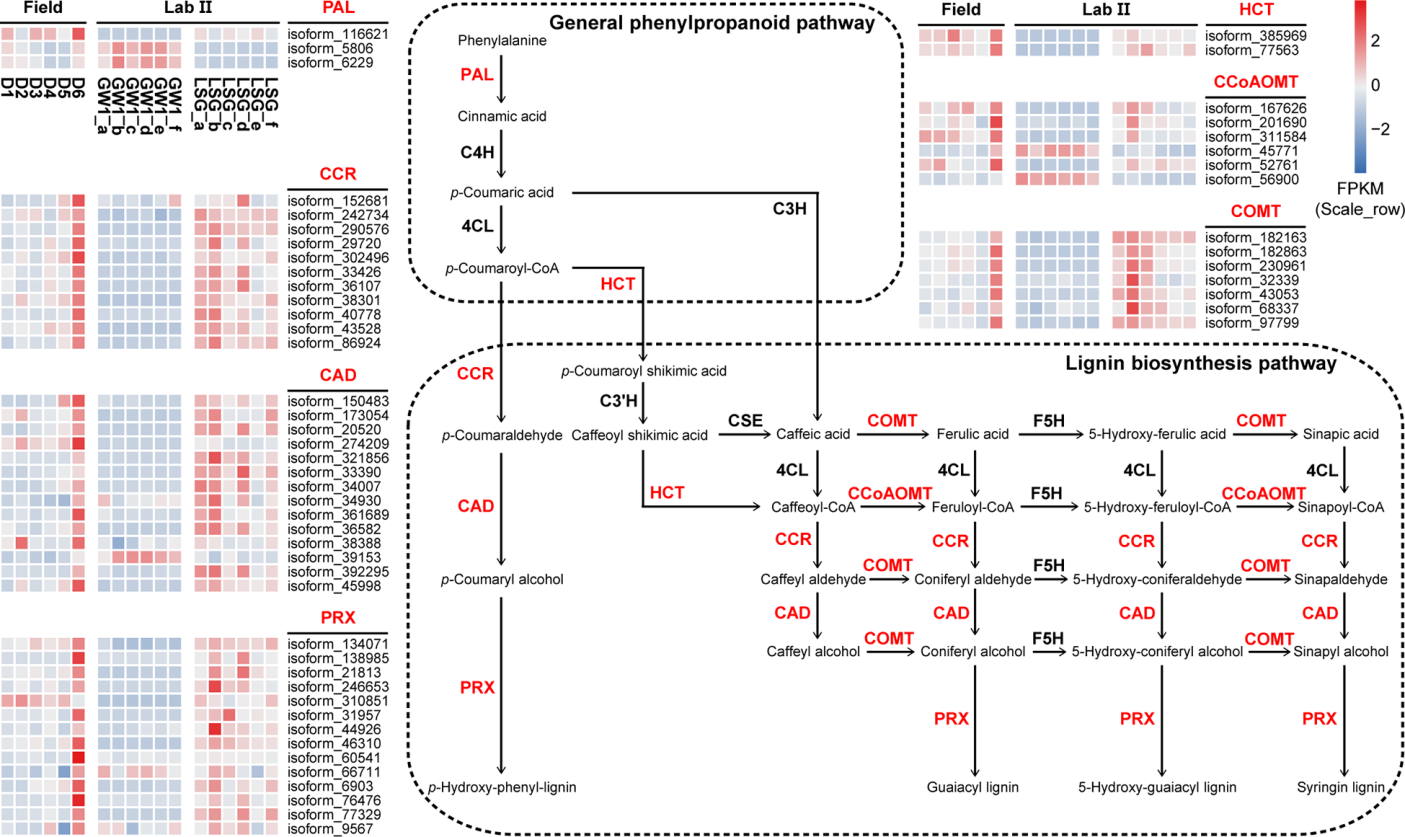
The lignin biosynthesis pathway in the ko00940 “phenylpropanoid biosynthesis” pathway and expression level cluster heat maps of key genes in this pathway in the field and lab experiments.

In the ko00940 “phenylpropanoid biosynthesis” pathway, 70 genes were divided into 11 functions, and 57 genes were annotated into the lignin biosynthesis pathway of this pathway, including K00083 “cinnamyl-alcohol dehydrogenase” (CAD) with 14 genes, K00430 “peroxidase” (PRX) with 14 genes, K09753 “cinnamoyl-CoA reductase” (CCR) with 11 genes, K13066 “caffeic acid 3-O-methyltransferase” (COMT) with 7 genes, K00588 “caffeoyl-CoA O-methyltransferase” (CCoAOMT) with 6 genes, K10775 “phenylalanine ammonia-lyase” (PAL) with 3 genes, and K13065 “shikimate O-hydroxycinnamoyltransferase” (HCT) with 2 genes (**Figure 7**). In the field experiment, most genes had low expression at D1, D2 and D3 before cold acclimation, some genes were up-regulated in D4 and D5 during cold acclimation, and most genes were up-regulated to the highest at D6 under freezing temperature; in the lab experiment II, the expression levels of most genes were higher in LSG samples than those in GW1 samples (**Figure 9**).

#### 3.7.3 Other KEGG pathways

In the ko00280 “valine, leucine and isoleucine degradation” pathway, 49 genes were divided into 6 functions, and 35 genes were annotated as K00020 “3-hydroxyisobutyrate dehydrogenase” (HIBADH) in this pathway (**Figure 7**). In the ko00902 “monoterpenoid biosynthesis” pathway, all 15 genes were annotated as K15095 “(+)-neomenthol dehydrogenase” (MND) (**Figure 7**). In the ko00480 “glutathione metabolism” pathway, 49 genes were divided into 6 functions, and 37 genes were annotated as K00799 “glutathione S-transferase” (GST) in this pathway (**Figure 7**). However, the upstream or downstream genes of HIBADH, MND and GST genes annotated in their respective pathways were rare. In the field experiment, most HIBADH, MND and GST genes showed the highest expression level at D6 under freezing temperature. In the lab experiment II, the expression level of most HIBADH, MND and GST genes were higher in LSG samples than those in GW1 samples.

## 4 Discussion

### 4.1 Field plus lab experiments for identifying freezing tolerance and associated genes

Based on a long research tradition, most plant biologists either focus on lab experiments or field experiments (Poorter et al., 2016). In fields, plants are affected by many environmental factors, and it is difficult to explore the responses of plants to a single factor, which often hinders a clear interpretation of field experimental data; in labs, although some of the experiments can be carried out under more controlled conditions, unfillable gaps remain between the responses of plants under controlled conditions in labs and the real situations in fields (Poorter et al., 2016; Hashida et al., 2022). Trying to fill such gaps in this study, we designed field plus lab experiments for identifying freezing tolerance and associated genes in evergreen broadleaf trees using *C. oleifera* as a case.

For the field experiment, we selected the high elevation site with naturally distributed wild *C. oleifera* in Lu Mountain because of the low freezing temperatures (<−10°C) in winter. Previous studies showed that wild *C. oleifera* may undergo cold acclimation when air temperature below 10°C (Chen et al., 2017), similar to *C. sinensis* (Wang et al., 2013). Therefore, depending on the continuously recorded air temperature in the field, the sampling periods were divided into three periods: 1) before cold acclimation (mean air temperature ≥10°C); 2) during cold acclimation (mean air temperature 0~10°C); and 3) under freezing temperature (mean air temperature <0°C) (**Figure 1A**). The field experiment may represent the actual responses of wild *C. oleifera* (e.g. LSG) to environmental changes in nature. However, various environmental factors other than air temperature may also be considerably different among the three periods resulting in different responses in wild *C. oleifera*. Thus, we also designed a lab experiment in a climate chamber with controlled freezing temperature (−10°C) to specifically evaluate the effects of freezing temperature on leaf samples of wild *C. oleifera* (LSG) from the third period. Leaf samples of a commonly cultivated *C. oleifera* (GW1) in the north Jiangxi Province was used as control in the lab experiment, supposing that cultivated *C. oleifera* may be more sensitive to freezing stress than wild *C. oleifera*. The relative conductivity (REC) (equation 1) was used to measure the responses of leaves to freezing temperature. We found that wild *C. oleifera* LSG samples was indeed more tolerant to freezing temperature than those of cultivated *C. oleifera* GW1, and the results were consistent in the lab experiments of two years (**Figures 2A, B**). Moreover, leaf samples before cold acclimation of both wild and cultivated *C. oleifera* suffered freeze injury soon after the freezing temperature treatment (**Figure 2E**). Such results indicated that for freezing tolerance evaluation samples should be taken after completing cold acclimation. However, trees usually could not complete cold acclimation under lab experimental conditions as shown by Liu et al. (2020). Thus, our study demonstrates that it is essential to combine field and lab experiments for evaluating freezing tolerance in perennial woody plants such as *C. oleifera*.

The gene expression patterns were also clearly differentiated among samples from the three periods in the field experiment, and between cultivated and wild *C. oleifera* samples from the lab experiment (**Figure 3**). Comparing genes correlated with the sample from the third period in the field experiment and the differentially expressed genes between cultivated and wild *C. oleifera* samples from the lab experiment (**Figure S9**), the common genes found in both of the field and lab experiments may be associated with the responses to freezing temperature. These genes represent about 66% of the genes correlated in the field experiment and only about 4% of the DEGs in the lab experiment. Such results indicated that neither field experiment nor lab experiment alone could represent the genes associated with the responses to freezing temperature. Again, our study demonstrates that it is essential to properly combine the genes found in the field and lab experiments for identifying genes associated with the responses to freezing temperature.

### 4.2 Differences in genes associated with cold acclimation and freezing temperature in the field experiment

The molecular mechanisms of cold acclimation have been widely studied in annual herbaceous plants mainly in labs (Strimbeck et al., 2015), supposing that the processes of cold acclimation provide solide supports to survive subsequent freezing temperature. However, annual herbaceous plants usully complete their life cycles before harder winters in nature. On the other hand, many perennial woody plants need to survive overwinter under extreme freezing temperature (Neuner, 2014). Thus, the molecular mechanisms of freezing tolerance in perennial woody plants may be far more different than those in annual herbaceous plants. Studies in perennial woody plants (e.g. *Vitis vinifera*) found that exposure to chill stress and freezing stress resulted in very dissimilar transcriptional landscapes (Londo et al., 2018). In this study, the results of the PCA and time-series analysis of the samples from the field experiment clearly demonstrated that the expression patterns of associated genes were very different during cold acclimation and under freezing temperature (**Figures 3A**, **S5**). In addition, we found that the functions of correlated genes were very different during cold acclimation and under freezing temperature (**Figure 5**).

During cold acclimation, many genes were found to be enriched in the “endocytosis” pathway with the highest level of significance in this study (**Figure 5A**). Endocytosis is important for signaling, stomatal movements and cell wall morphogenesis (Samaj et al., 2005). Studies showed that endocytosis related proteins increase during cold acclimation in Arabidopsis (Minami et al., 2009). In *Zanthoxylum bungeanum*, endocytosis related genes were found to be at the core of the regulatory network during cold acclimation (Tian et al., 2021). Moreover, some genes were very highly significantly enriched in the “fatty acid elongation” pathway or “plant hormone signal transduction” pathway during cold acclimation (**Figure 5A**). The former plays an important role in fatty acid biosynthesis (Haslam and Kunst, 2013), which may help maintain cell membrane stability to mitigate cell damage; the latter may be closely related to the cold signal transduction, contributing to the productions of various protective substances in cold stress (Shi et al., 2015). Most of the genes significantly enriched in these pathways were up-regulated to the highest expression levels during cold acclimation (**Figure S7**).

Under freezing temperature, more genes were found to be enriched in the “plant hormone signal transduction” pathway with a higher level of significance than during cold acclimation (**Figure 5**). Moreover, under freezing temperature, much more genes were found to be very highly significantly enriched in very different pathways from cold acclimation (**Figure 5**). These enriched pathways have also been found involving in responses to freezing stress in previous studies, such as the “MAPK signaling pathway-plant” pathway for cold signal transduction (Guo et al., 2018), the “plant-pathogen interaction” pathway for defense responses (Zarattini et al., 2021; Jin et al., 2022), and the “phenylpropanoid biosynthesis” pathway for the synthesis of important secondary metabolites (Dong and Lin, 2021). Most of the genes significantly enriched in these pathways were up-regulated to the highest levels of expression under freezing temperature in this study (**Figure S7**). However, being compared with the state of the art of the molecular mechanisms of cold acclimation, the molecular responses under freezing temperature are lacking in-depth studies especially in perennial woody plants.

### 4.3 Important roles of flg22, Ca^2+^ and gibberellin signal transduction pathways in freezing tolerance

A recent study showed that pathways activated by flg22 could not only help plants defend against pathogen infection, but also induce expression of cold tolerance related genes to alleviate cold injury and enhance cold tolerance (Jin et al., 2022). In plant cells, flg22 is recognized by FLS2, and activates a mitogen-activated protein kinase (MAPK) cascade to promote the expression of downstream related genes (Zipfel et al., 2004), such as WRKY33 and PR1. In our study, most FLS2, MEKK1, MPK3, VIP1, PR1 and WRKY33 genes in the flg22 signal transduction pathway were up-regulated to the highest expression levels under freezing temperature in the field experiment, and in the lab experiment these genes showed higher expression levels in LSG samples with stronger freezing tolerance than in GW1 samples (**Figure 8**). Many genes were annotated as WRKY33 in the flg22 signal transduction pathway (**Figure 7**). WRKY33 is one of the most widely studied members of the WRKY family. A recent study found that the expression levels of WRKY33 genes were almost unchanged in cold-sensitive tomato but were significantly induced in cold-tolerant tomato under 4°C; silencing the WRKY33 gene decreased cold tolerance of tomato seedlings and overexpressing the WRKY33 gene enhanced cold tolerance of tomato seedlings (Guo et al., 2022). Thus, the up-regulations of the WRKY33 genes may also be associated with the freezing tolerance in wild *C. oleifera*. Additionally, PR proteins can be converted into antifreeze proteins with antifreeze activity in cold stress for inhibiting ice crystal growth (Gupta and Deswal, 2014). Therefore, the high expression of PR1 genes under freezing temperature in our study may also help enhance freezing tolerance in wild *C. oleifera*.

In this study, WRKY33 genes may also be activated in the Ca^2+^ signal transduction pathway under freezing temperature. Moreover, some other genes (CNGC, CaM, CML, CDPK and Rboh) in the Ca^2+^ signal transduction pathway were also found to be up-regulated under freezing temperature in both of the field and lab experiments (**Figure 8**). As a crucial second messengers, Ca^2+^ are involved in the regulation of numerous cellular functions (Berridge et al., 2000). When plants are stimulated by biotic or abiotic stresses, the cytosolic Ca^2+^ concentration in plants will increase due to Ca^2+^ influx (Yang and Poovaiah, 2003). Calcium channels such as CNGC, contribute to Ca^2+^ influx in the responses to various stresses. In rice, the expression levels of CNGC genes were significantly up-regulated during 4°C treatment, indicating that CNGC actively participated in the response to chilling stress (Nawaz et al., 2014). Similarly, the CNGC gene showed the highest expression level under freezing temperature and higher expression levels in LSG samples with stronger freezing tolerance than in GW1 samples (**Figure 8**). The increase in cytosolic Ca^2+^ concentration may activate Ca^2+^ sensors such as CaM, CML and CDPK (Ranty et al., 2016) to transmit Ca^2+^ signal, which may activate the expression of downstream Rboh genes (**Figure 8**). Rboh are important enzymes inducing ROS productions in plant growth, development, responses to environmental signals (Suzuki et al., 2011), and regulation of stomatal closure (Chapman et al., 2019). Many cis-acting elements associated with stress responses such as light, low/high temperature and drought were identified in the Rboh genes of *Arabidopsis thaliana* and rice (Kaur and Pati, 2016).

As an important hormone, gibberellin plays regulatory roles in plant growth, development and reproduction (Davies, 1995). In recent years, many studies have confirmed that gibberellin is also involved in plant tolerance to abiotic stress (Jiang et al., 2016). The biological roles of gibberellin in plants are achieved through the gibberellin-GID1-DELLA signaling transduction pathway. In general, gibberellin levels decrease with the decrease of temperature, and DELLA accumulates, leading to slow growth of plants (Kurepin et al., 2013). Studies have shown that DELLA affects the stress tolerance of plants, and the more DELLA the stronger tolerance to stress (Huang et al., 2006). In our study, many genes were annotated as DELLA genes in the gibberellin signal transduction pathway. Most of the DELLA genes were up-regulated under freezing temperature in the field experiment and showed higher expression levels in LSG samples with stronger freezing tolerance than in GW1 samples in the lab experiment (**Figure 8**). Such results suggested that the up-regulation of the DELLA genes were also associated with freezing tolerance in wild *C. oleifera*.

### 4.4 Lignin biosynthesis and freezing tolerance

“Phenylpropanoid biosynthesis” pathway is one of the most important secondary metabolic pathways in plants and plays a crucial role in plant development and responses to stresses (Dong and Lin, 2021). In most plants, “phenylpropanoid biosynthesis” pathway begins with phenylalanine being produced *via* shikimate pathway, and then phenylalanine is catalyzed by PAL, C4H and 4CL, constituting the general phenylpropanoid pathway (Fraser and Chapple, 2011). The lignin biosynthesis pathway is one of the main branches downstream the general phenylpropanoid pathway. The lignin biosynthesis processes are accomplished by an orchestrated cascade of enzymes, such as HCT, COMT, CCoAOMT, CCR, CAD and PRX, with functions including acylation, methylation, glycosylation, and hydroxylation (Dong and Lin, 2021). As one of the main components of plant cell wall, lignin constitutes the support and transport systems of plants and affects the ability to tolerate biotic or abiotic stresses (Liu et al., 2018b; Han et al., 2022). Although herbaceous and woody plants have similar lignin biosynthesis pathways, the differences in temporal and spatial expression patterns of genes in the lignin biosynthesis pathways may lead to differences in stress tolerance between them (Han et al., 2022). In barley leaves, the expressions of the lignin biosynthesis related genes including PAL, HCT and CAD genes were up-regulated during cold acclimation (Janska et al., 2011). Based on transcriptome sequencing of leaf samples from 60-year-old overwintering *Camellia sinensis*, the expressions of PAL, CCR, HCT and COMT genes related to lignin biosynthesis in the “phenylpropanoid biosynthesis” pathway were found to be significantly up-regulated under freezing stress, indicating that lignin biosynthesis was associated with freezing tolerance in *C. sinensis* (Wu et al., 2021).

In our study, most genes in the lignin biosynthesis pathway were annotated, including PAL, CCR, HCT, CAD, PRX, CCoAOMT and COMT genes (**Figure 9**). Most of these genes had the highest expression levels under freezing temperature in the field experiment. In the lab experiment, the expression levels of these genes were higher in LSG samples with stronger freezing tolerance than in GW1 samples under freezing temperature (**Figure 9**). Such results suggested that the up-regulations of the genes in the lignin biosynthesis pathway were also associated with the freezing tolerance in wild *C. oleifera*.

## 5 Conclusion

**Figure 10:**
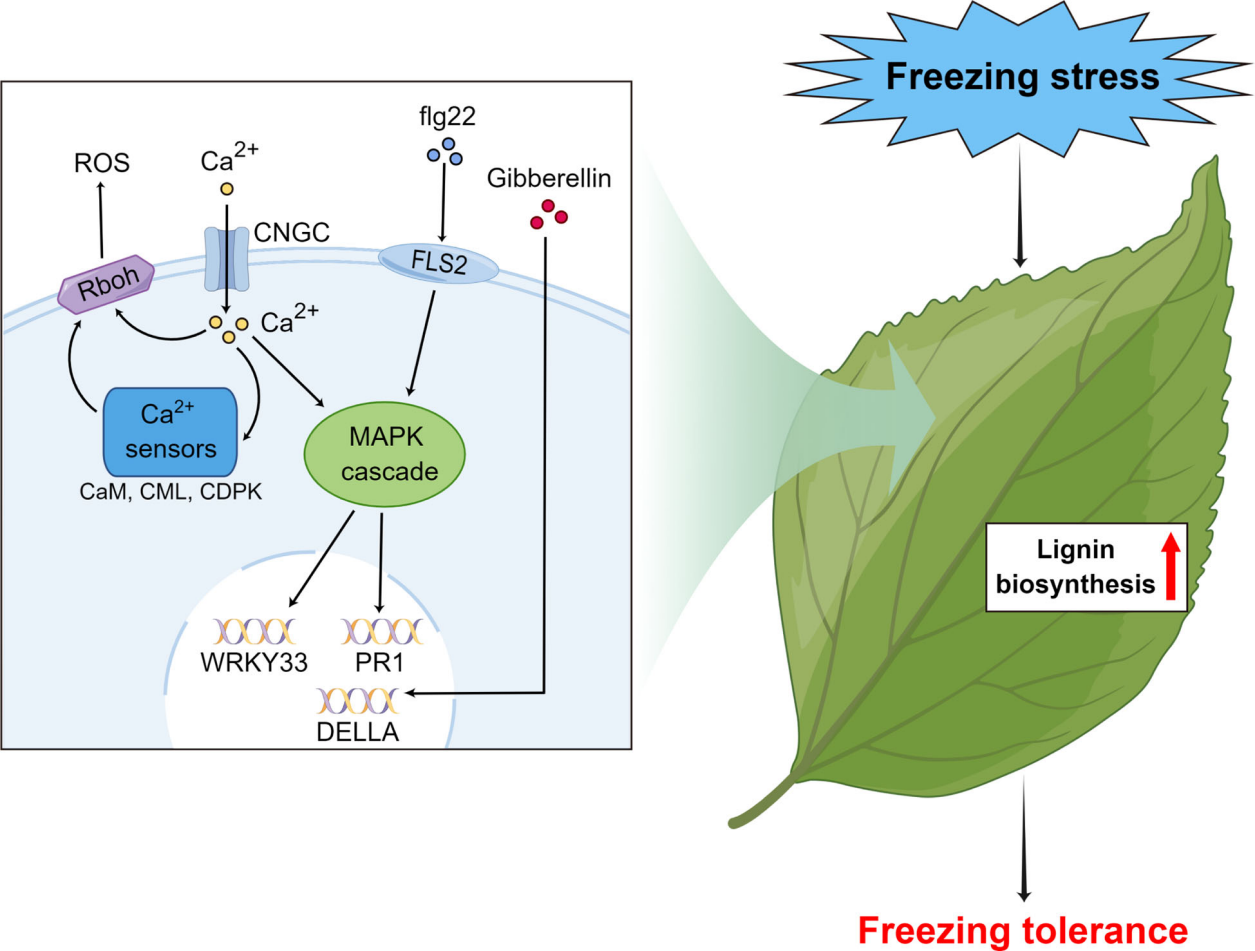
Hypothetical model of the important pathways and key genes associated with responses to freezing stress in subtropical evergreen broadleaf trees such as *Camellia oleifera* made by Figdraw.

Our study proposes a method using field plus lab experiments to identify freezing tolerance and associated genes in subtropical evergreen broadleaf trees. As a case, we found that wild *C. oleifera* from the high elevation site in Lu Mountain was indeed more freezing tolerant than a commonly cultivated *C. oleifera*. For freezing tolerance evaluation, samples should be taken after completing cold acclimation. The transcriptome analyses of leaf samples from the field experiment showed that the gene functions and expression patterns were very different during cold acclimation and under freezing temperature. By combining transcriptome sequencing results of leaf samples under freezing temperature in the field and lab experiments, genes associated with freezing tolerance were identified in wild *C. oleifera*. We propose a hypothetical model for the molecular mechanisms of freezing tolerance in wild *C. oleifera* (**Figure 10**). The flg22, Ca^2+^ and gibberellin signal transduction pathways actively participate in the responses to freezing stress and some key genes of the pathways play important roles, such as WRKY33, PR1, Rboh and DELLA genes. Moreover, most of the genes in the lignin biosynthesis pathway may also play important roles in freezing tolerance. Most of the key genes had the highest expression levels under freezing temperature in the field experiment and showed higher expression in wild *C. oleifera* with stronger freezing tolerance under freezing temperature in the lab experiment. Our study may facilitate the exploration of genetic resources with freezing tolerance and help understand the underlying molecular mechanisms in perennial woody plants.

## Data availability statement

The datasets presented in this study can be found in online repositories. The name of the repository and accession number can be found below: NCBI Sequence Read Archive, PRJNA915196.

## Author contributions

HX, JZ, and JR designed the experiments. HX conducted the experiments with help from JZ, JC, SZ, QW, and JR. HX, JZ, PK, YZ, XX, and JR participated in data analyses. All authors contributed to the article and approved the submitted version.
