# Supplementary material for: Field plus lab experiments help identify freezing tolerance and associated genes in subtropical evergreen broadleaf trees: A case study of Camellia oleifera
Source: Front Plant Sci. 2023 Feb 22;14:1113125. doi: 10.3389/fpls.2023.1113125 (PMC9994817; doi:10.3389/fpls.2023.1113125)
Supplement: Supplementary file 1 [file DataSheet_1.pdf]

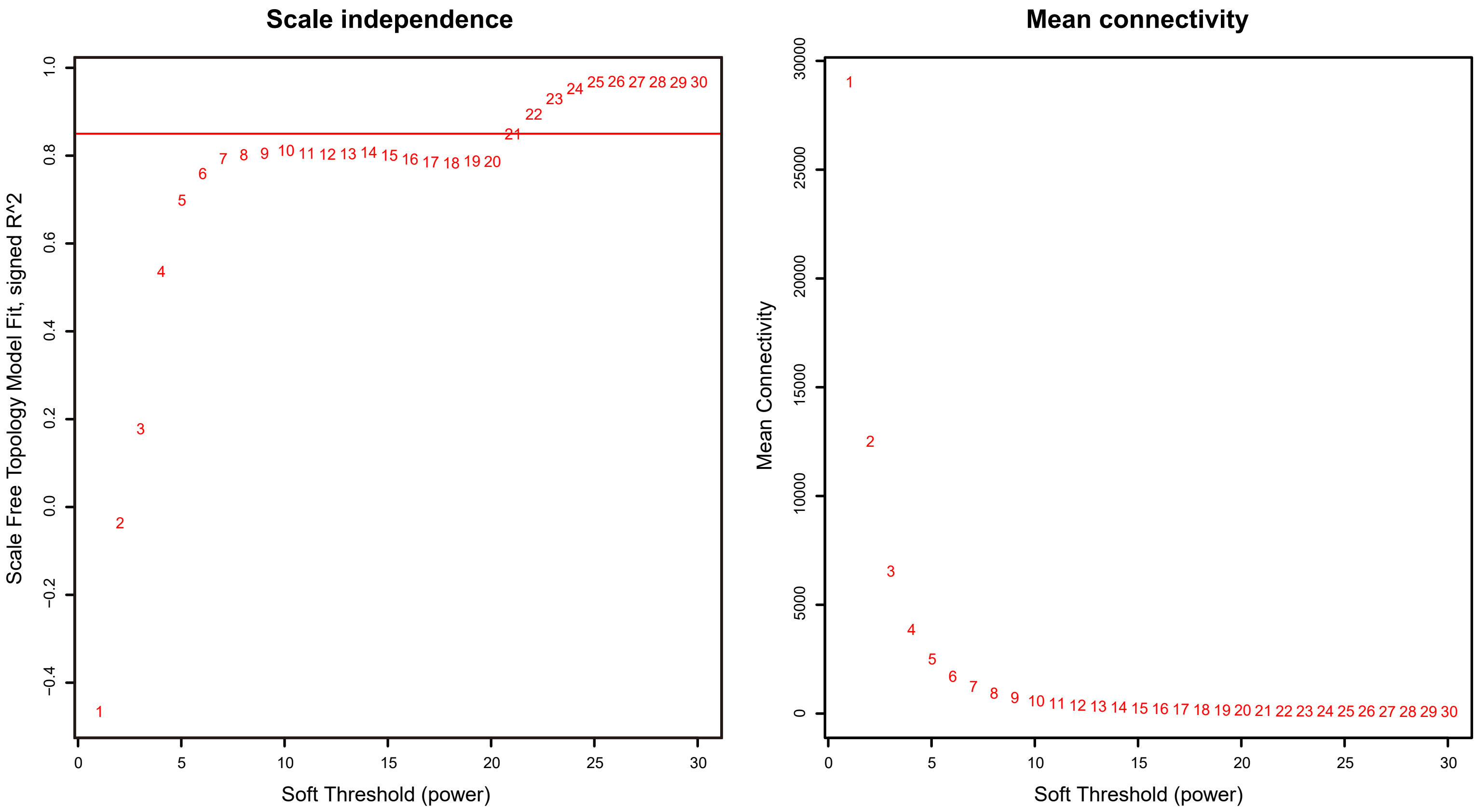

Figure S1. Soft power plot. The abscissa represents the soft threshold, the ordinate represents the scale-free topology model fit index (left), and the mean connectivity index (right).

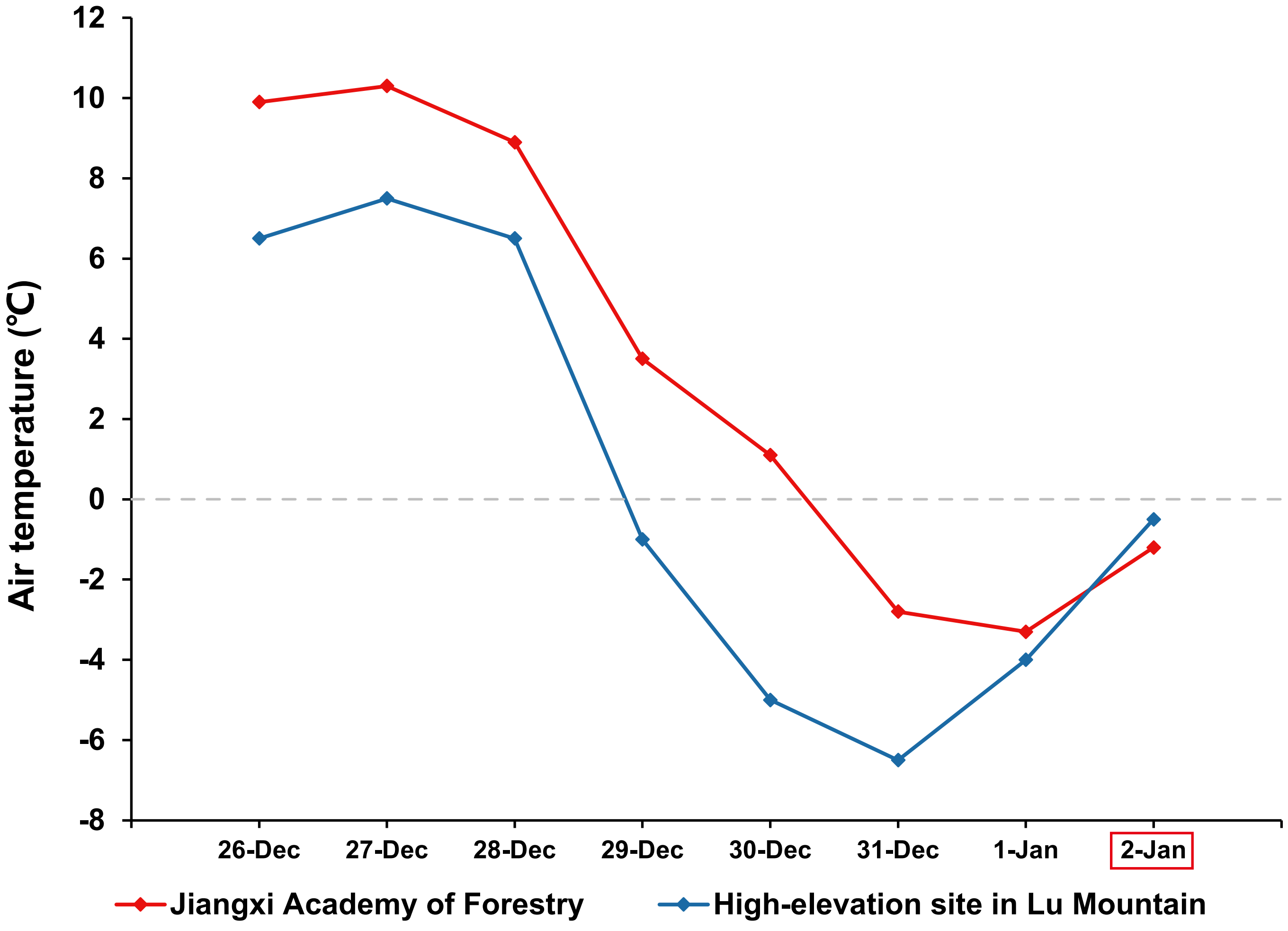

Figure S2. Minimum temperatures at Jiangxi Academy of Forestry and high-elevation site in Lu Mountain from December 26, 2020 to January 2, 2021. Samples were collected on Jan 2 (D6 in the field experiment) and used in the lab experiment II.

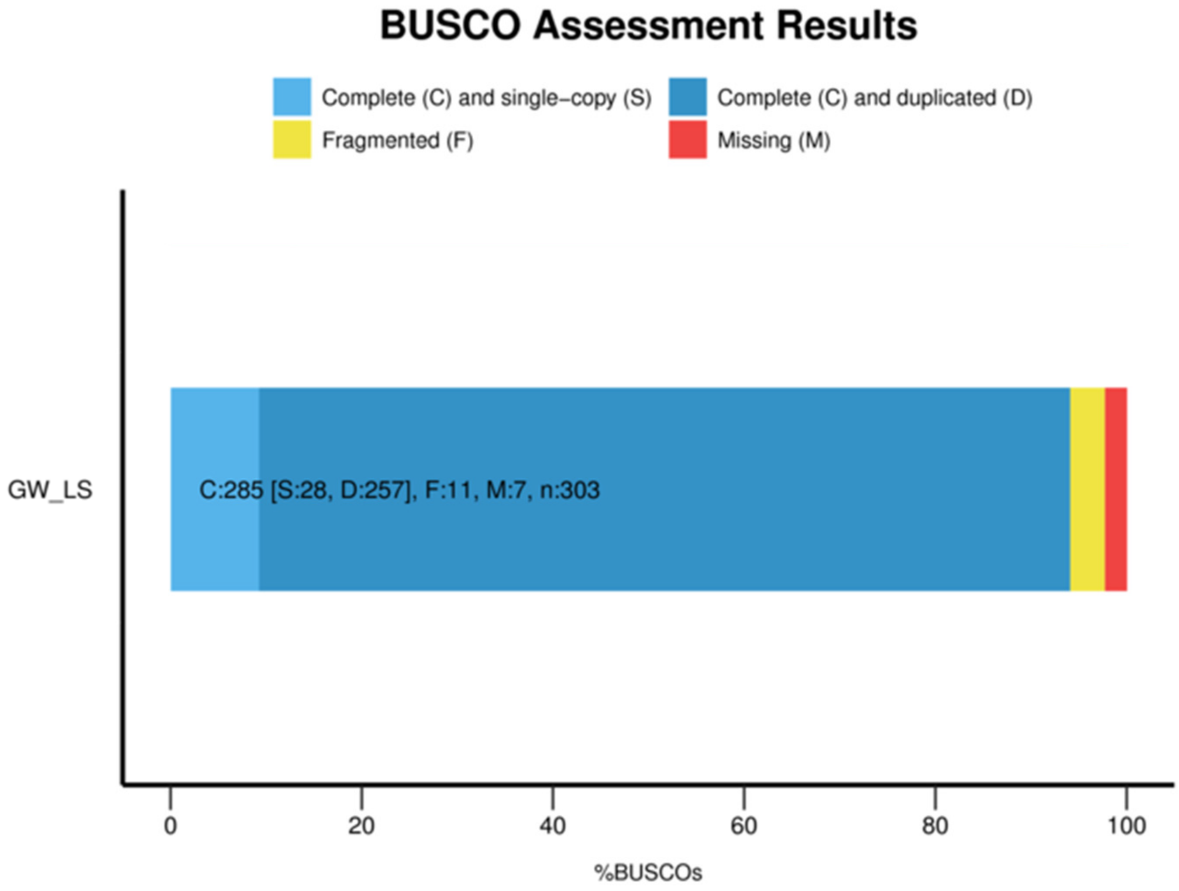

Figure S3. Quality assessment results of the ISO-seq data.

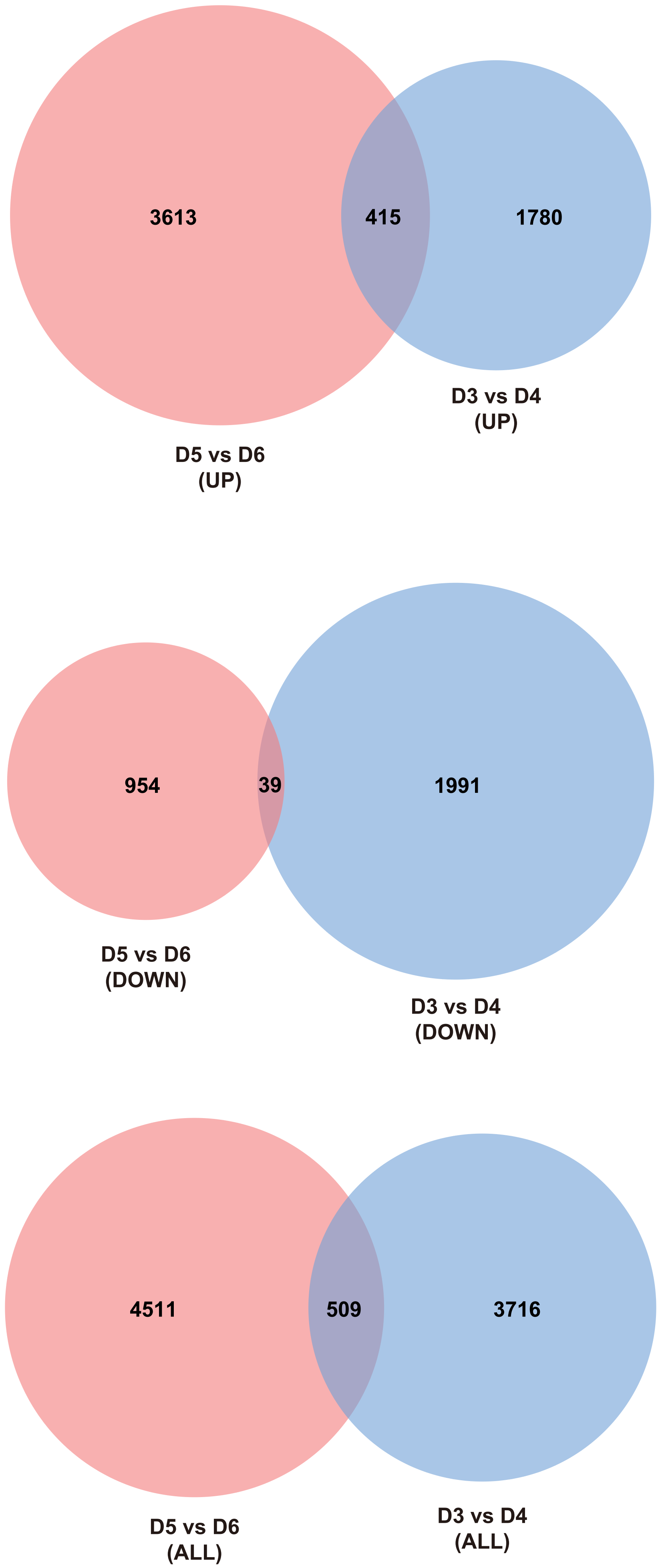

Figure S4. Venn diagrams of differentially expressed genes between samples at D3 and D4, and samples at D5 and D6 in the field experiment.

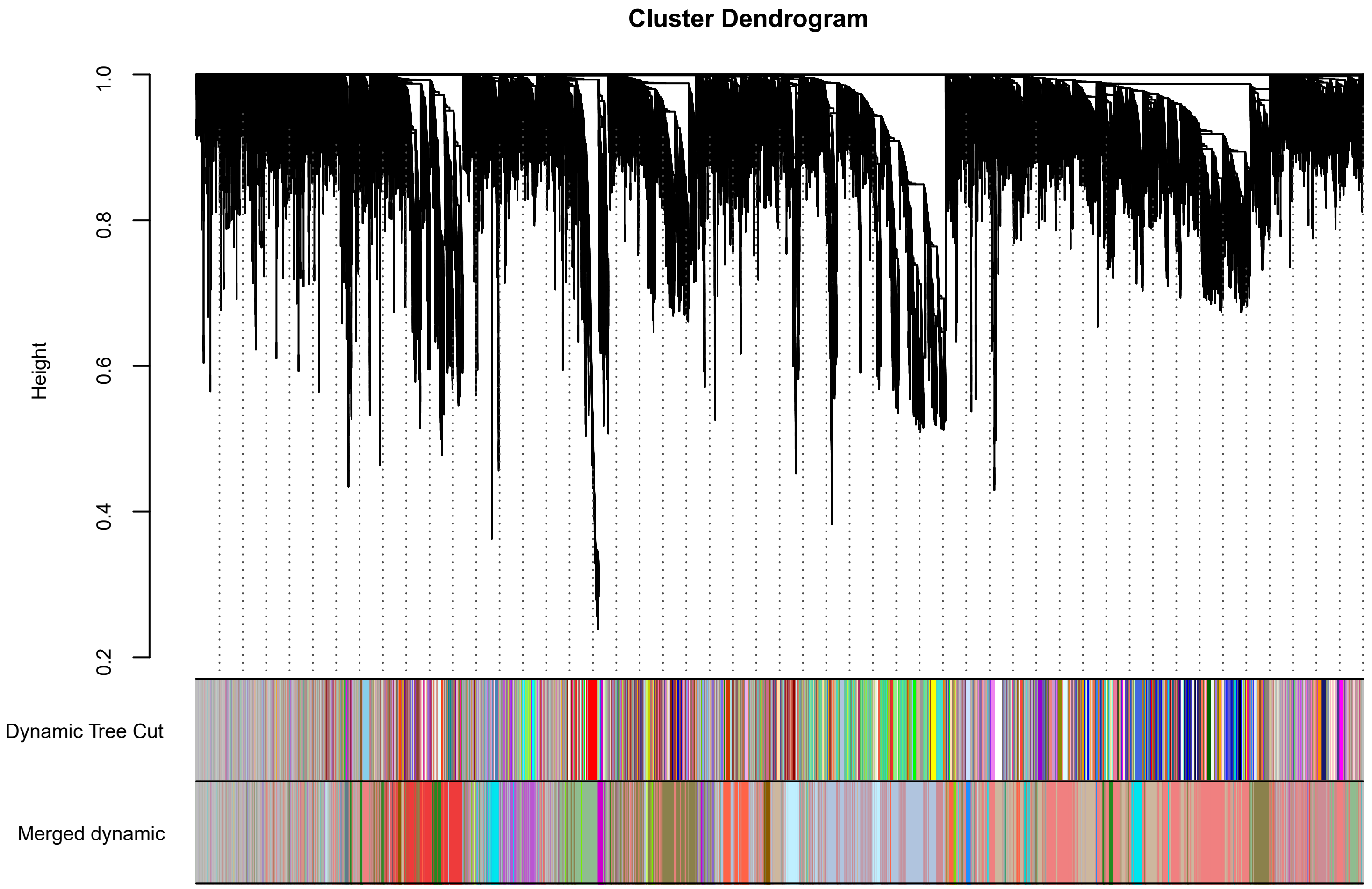

Figure S5. Clustering dendrogram of genes and module division. The dynamic tree cut represents the module divided according to the expression of each gene, while the merged dynamic is the result of merging similar modules according to the dynamic tree cut.

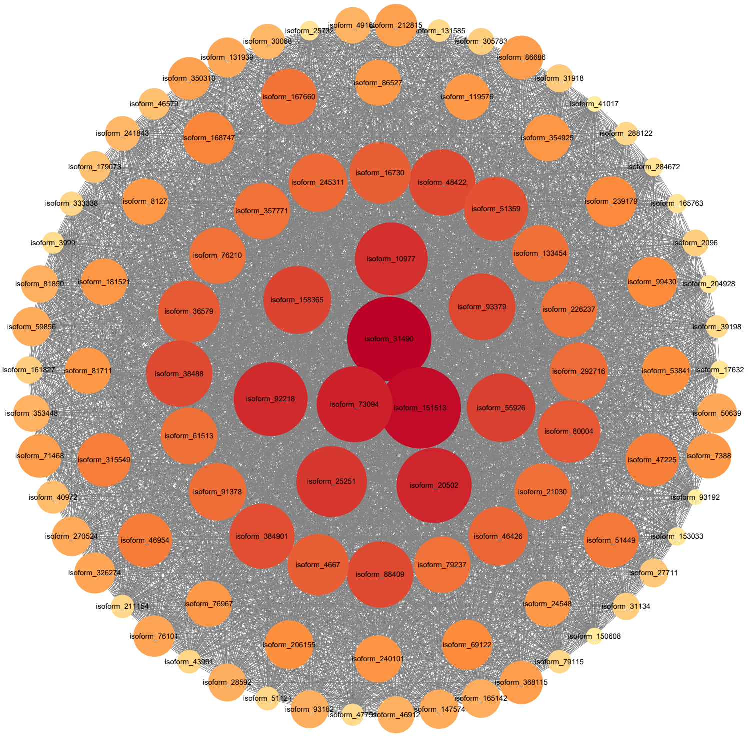

Figure S6. Co-expression network of the top 100 hub genes from the ME_brown2 module. Node size and node color depth represents connectivity (Degree).

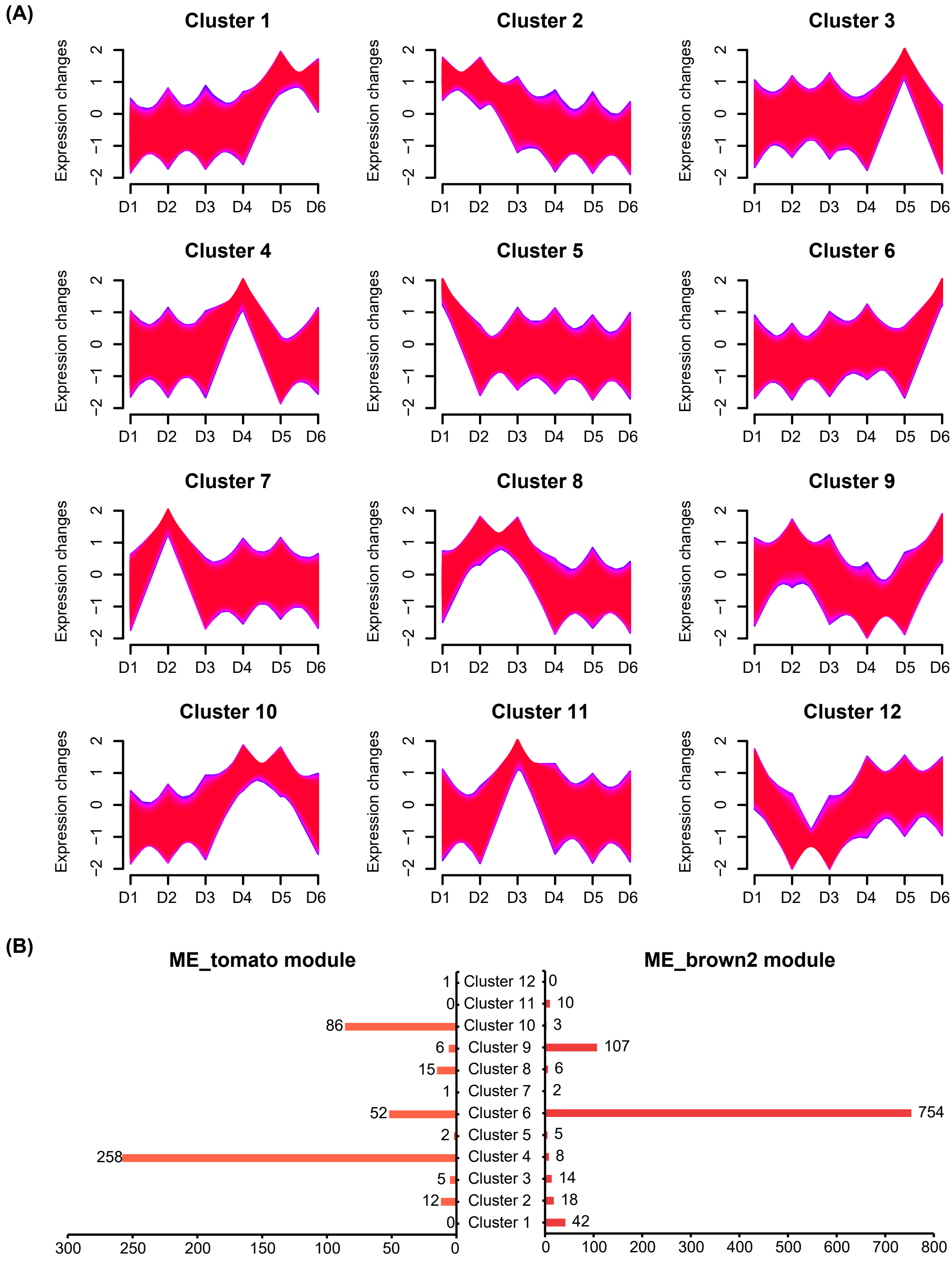

Figure S7. (A) Time-series analysis based on the FPKM data of all genes in the field experiment. (B) The number of genes significantly enriched in the KEGG pathway from the ME_tomato module and the ME_brown2 module in each cluster.

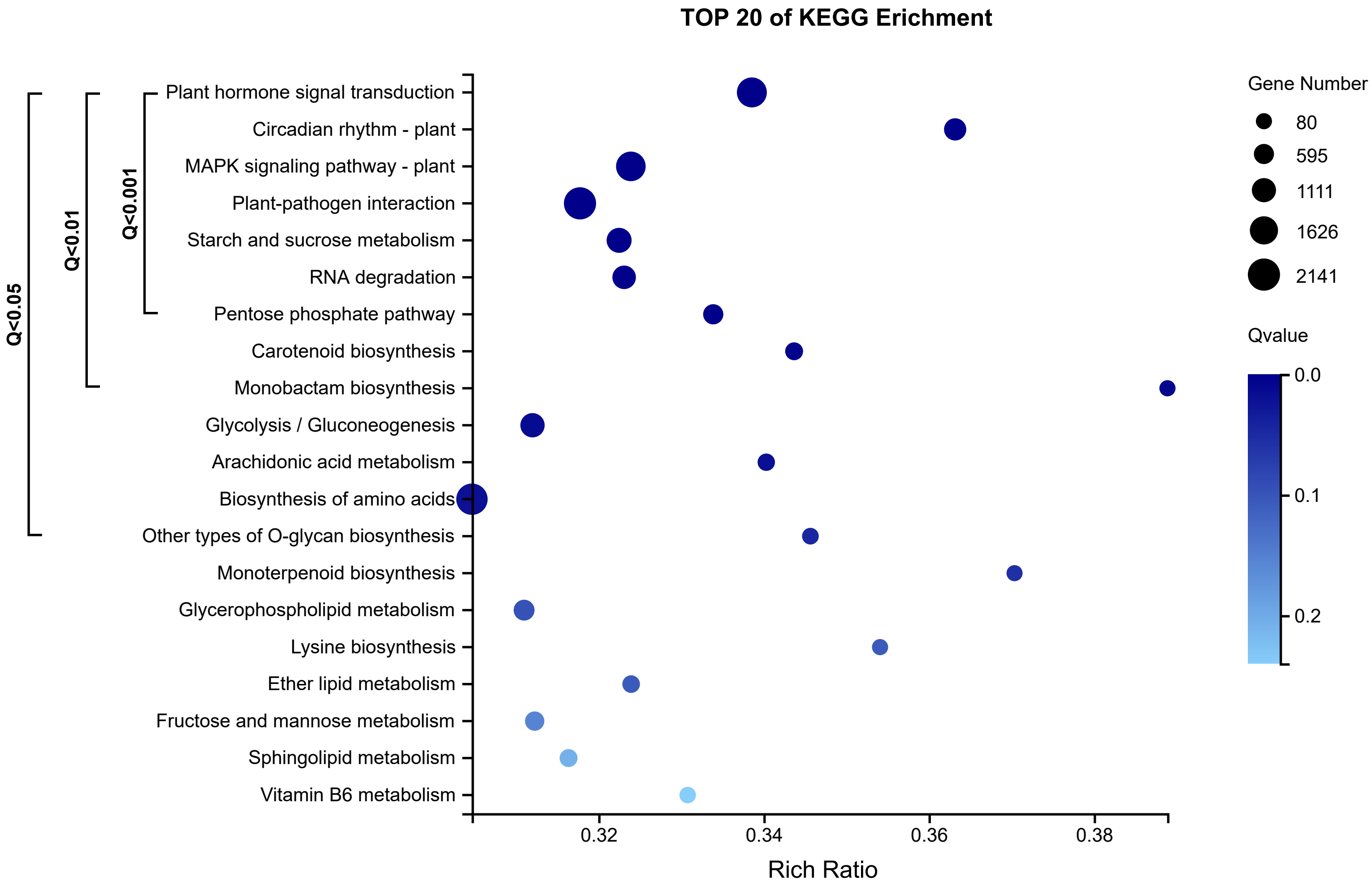

Figure S8. KEGG pathway enrichment results of 88290 DEGs between GW1 and LSG samples at −10°C in the lab experiment II.

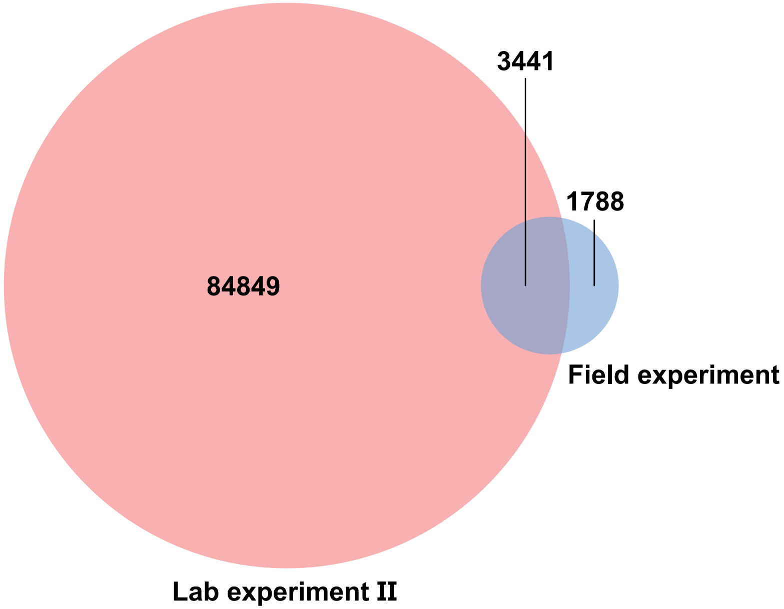

Figure S9. Venn diagram of genes in the ME_brown2 module from the field experiment and the DEGs between GW1 and LSG samples from the lab experiment II.
